# Distinct pathway-based effects of blood pressure and body mass index on cardiovascular traits: comparison of novel Mendelian randomization approaches

**DOI:** 10.1186/s13073-025-01472-2

**Published:** 2025-05-15

**Authors:** Genevieve M. Leyden, Maria K. Sobczyk, Tom G. Richardson, Tom R. Gaunt

**Affiliations:** MRC Integrative Epidemiology Unit, Bristol Medical School, University of Bristol, Oakfield House, Bristol, BS8 2BN UK

**Keywords:** Body mass index, Blood pressure, Cardiovascular disease, Mendelian disease, Colocalization, Mendelian randomization, Genetic epidemiology

## Abstract

**Background:**

Mendelian randomization (MR) leverages trait associated genetic variants as instrumental variables (IVs) to determine causal relationships in epidemiology. However, genetic IVs for complex traits are typically highly heterogeneous and, at a molecular level, exert effects on different biological processes. Exploration of the biological underpinnings of such heterogeneity can enhance our understanding of disease mechanisms and inform therapeutic strategies. Here, we introduce a new approach to instrument partitioning based on enrichment of Mendelian disease categories (pathway-partitioned) and compare it to an existing method based on genetic colocalization in contrasting tissues (tissue-partitioned).

**Methods:**

We employed individual- and summary-level MR methodologies using SNPs grouped by pathway informed by proximity to Mendelian disease genes affecting the renal system or vasculature (for blood pressure (BP)), or mental health and metabolic disorders (for body mass index (BMI)). We compared the causal effects of pathway-partitioned SNPs on cardiometabolic outcomes with those derived using tissue-partitioned SNPs informed by colocalization with gene expression in kidney, artery (BP), or adipose and brain tissues (BMI). Additionally, we assessed the likelihood that estimates observed for partitioned exposures could emerge by chance using random SNP sampling.

**Results:**

Our pathway-partitioned findings suggest the causal relationship between systolic BP and heart disease is predominantly driven by vessel over renal pathways. The stronger effect attributed to kidney over artery tissue in our tissue-partitioned MR hints at a multifaceted interplay between pathways in the disease aetiology. We consistently identified a dominant role for vessel (pathway) and artery (tissue) driving the negative directional effect of diastolic BP on left ventricular stroke volume and positive directional effect of systolic BP on type 2 diabetes. We also found when dissecting the BMI pathway contribution to atrial fibrillation that metabolic-pathway and brain-tissue IVs predominantly drove the causal effects relative to mental health and adipose in pathway- and tissue-partitioned MR analyses, respectively.

**Conclusions:**

This study presents a novel approach to dissecting heterogeneity in MR by integrating clinical phenotypes associated with Mendelian disease. Our findings emphasize the importance of understanding pathway-/tissue-specific contributions to complex exposures when interpreting causal relationships in MR. Importantly, we advocate caution and robust validation when interpreting pathway-partitioned effect size differences.

**Supplementary Information:**

The online version contains supplementary material available at 10.1186/s13073-025-01472-2.

## Background

Mendelian randomization (MR) is a statistical method used in epidemiology to study the causal relationship between a risk factor (exposure) and an outcome (disease or trait) by leveraging genetic variants derived from genome-wide association studies (GWAS) as instrumental variables [1] (IV). The technique is based on the principles of Mendelian inheritance, which states that genetic variants, such as single nucleotide polymorphisms (SNPs), are randomly assigned during meiosis and therefore should be less prone to confounding factors or reverse causation that typically plague observational studies (subject to meeting certain assumptions [2]).

With the availability of highly powered GWAS for complex traits, we are presented with large numbers of trait-associated SNPs from which to select genetic IVs of the exposure, which can yield variable causal estimates [3]. There are various sources of heterogeneity which violate the assumptions of MR and should be avoided when selecting instrumental variables because they can induce bias in the causal effect. These include (1) horizontal pleiotropy, which occurs when one or more SNPs influence the outcome through multiple independent pathways, and (2) weak instrument bias, which can result in imprecise causal estimates and increase heterogeneity [4]. Of chief interest in this study is heterogeneity arising due to endpoint phenotypes being de facto composites, representing divergent underlying biological mechanisms captured by different genetic instruments. Unlike the sources of bias mentioned above, leveraging this source of genetic heterogeneity in IV selection can improve understanding of disease aetiology and help design better targeted interventions.

Three broad types of approaches have been used so far when studying biological sources of heterogeneity in MR: direct clustering based on SNP associations with exposure and outcome [5, 6], clustering of variant associations across a set of traits [7–9], or instrument clustering informed by tissue gene expression patterns [10–13]. In particular, a biological hypothesis-driven approach proposed in Leyden et al. [11] clusters genetic instruments for body mass index (BMI) based on the tissue (brain or subcutaneous adipose) where a given BMI SNP is found to colocalize with an expression quantitative trait locus (eQTL). The Bayesian colocalization method coloc [14] is employed here to compare the association signals at a specific genomic region for the two traits of interest (gene eQTL and BMI). In this way, a given genetic instrument is putatively linked to a particular gene whose expression (either in subcutaneous adipose or brain tissue, or both) potentially contributes differentially to a set of cardiometabolic traits.

Although this approach was used to prioritize the putative causal tissue types underlying BMI-associated genes, in general the coloc method as originally implemented has been shown to lack specificity when assigning SNPs to genes on its own, particularly when using eQTL data due to the co-expression of nearby genes [15]. Another approach of prioritizing candidate genes at GWAS loci is to leverage the knowledge of Mendelian monogenic diseases, which are caused by rare mutations with large effects on phenotypes. Several studies have reported an enrichment of Mendelian disease genes near GWAS loci across various phenotypes, suggesting shared genetic basis between complex and Mendelian traits [16–18]. While not all Mendelian disease genes are equally relevant for a given complex trait, the alignment of genetic associations to shared phenotypes or symptoms of monogenic and complex forms of disease is a key metric for gene prioritization [19].

In this paper, we introduce a new approach to stratifying and annotating genetic instruments for common complex exposures used in MR informed by Mendelian disease categories. Blood pressure (BP) is a highly polygenic risk factor for a number of cardiovascular [20–22] and metabolic [23–25] conditions, with both vasculature- [26–28] and kidney- [29–31] expressed genes shown previously to be of key importance. Kidneys control blood pressure by regulating blood volume and electrolyte balance [32], chiefly through natriuresis response [33] and the renin–angiotensin–aldosterone system (RAAS) hormonal axis. Accordingly, impaired kidney function has long been linked to hypertension [34]. The vasculature regulates blood pressure via modulation of vascular tone. This is achieved through the processes of vasoconstriction and vasodilation, controlled by the smooth muscle cells in the arterial walls [35]. The endothelium lining the inner surface of blood vessels plays a pivotal role by releasing an array of vasoactive substances [36]. Endothelial cells secrete endothelin, a potent vasoconstrictor, and nitric oxide, the key vasodilator [37, 38], and can also influence blood pressure through inflammatory mechanisms [39]. If instruments acting on BP via these two key mechanisms show different estimates of effect on an outcome in MR, we hypothesize that this will be due to distinct biological effects represented in one or both subsets of instruments.

**Fig. 1:**
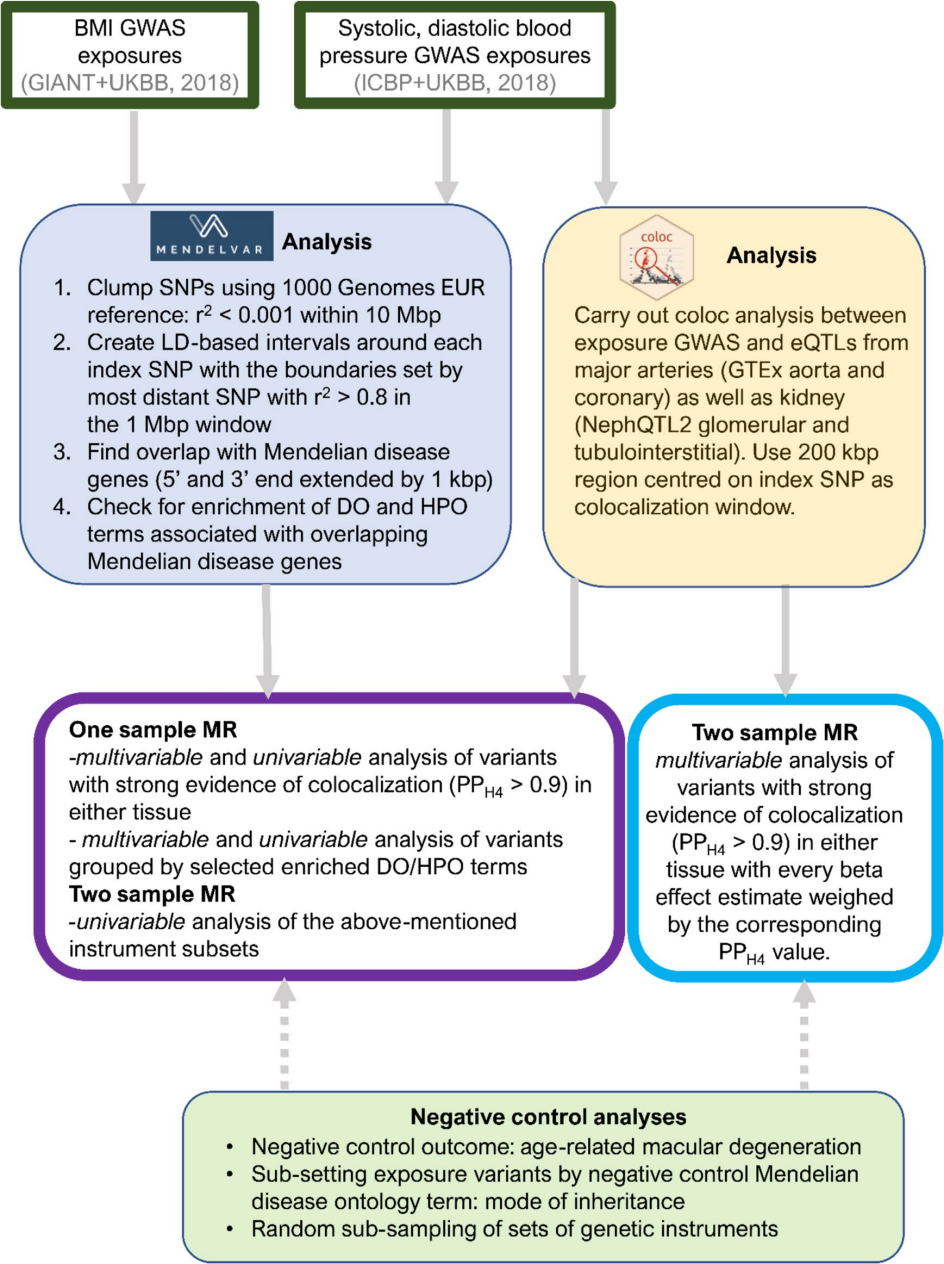
An overview of this study’s workflow for Mendelian disease gene pathway-partitioned genetic instruments and colocalization-derived tissue-partitioned genetic instruments with the aim of investigating pathway-specific effects of blood pressure and body mass index on cardiometabolic traits

We begin by contrasting the kidney and vascular components of blood pressure burden on cardiometabolic disease. To achieve this, we carry out one-sample and two-sample multivariable MR analyses utilizing blood pressure (systolic and diastolic) exposure variants grouped by co-sharing genetic loci with Mendelian disease genes whose symptoms affect either the renal system or vasculature (i.e. pathway effects) (Fig. 1). We compare our results to the colocalization-based method proposed previously [11, 12] by linking blood pressure genetic variants to regulation of gene expression in kidney or arteries (tissue effects). We then return to the BMI exposure reported by [12]to ask if the effect of variants grouped by metabolic and mental health Mendelian disease corresponds to the effects obtained by subcutaneous adipose and brain tissue. The comparison of both methods provides complementary insight on the genetic signals underlying symptomatic effects on the system (using the pathway approach) or molecular effects originating from the tissue (using the tissue approach). Finally, using random re-sampling of the genetic IVs of the exposure, we investigate if the effect size differences observed in pathway- or tissue-partitioned MR analyses are likely to have arisen by chance. Our findings provide valuable insights into the biological underpinnings of causal links between BP or BMI and cardiometabolic traits.

## Methods

### Exposure and outcome GWAS datasets

Our exposure datasets consisted of the most highly powered meta-analysed GWAS summary statistics at the time of analysis. The systolic (SBP) and diastolic blood pressure (DBP) GWAS (*N*: 757,601) undertaken by ICBP in 2018 [27] and body mass index (BMI) conducted by the GIANT consortium in 2018 (*N*: 681,275) [40] (Additional file 1: Table S1). These large studies, comprising ~ 0.7 million individuals of European ancestry respectively, were highly powered and returned a number of top variants (~ 900) implicated in a variety of biological processes in each GWAS.

Our outcome GWAS datasets included common cardiometabolic diseases: atrial fibrillation (AF) (*N* cases/controls (c/c): 60,620/970,216) [41], heart failure (HF) (*N* c/c: 47,309/930,014) [42], coronary heart disease (CHD) (*N* c/c: 60,801/123,504) [43], myocardial infarction (MI) (*N* c/c: 43,676/128,199) [43], stroke (*N* c/c: 40,585/406,111) [44], and type 2 diabetes (T2D) (*N* c/c: 12,171/56,862) (see Additional file 1: Table S1 for further details). GWAS for continuous measurements of cardiac function included left ventricular end-diastolic volume (LVEDV), left ventricular end-systolic volume (LVESV), left ventricular ejection fraction (LVEF), and left ventricular stroke volume (SV), all *N*: 36,041 UK Biobank participants [45]. As a negative control, we selected an outcome whose incidence is likely not to be causally impacted by BMI or BP as indicated by a previous MR study [46]: age-related macular degeneration (AMD) [46] (*N* c/c: 14,034/91,214).

We note that we used exposure and outcome GWAS datasets with non-overlapping participants whenever feasible, to limit bias [47], however a significant proportion of individuals in the AF and early-onset AMD (~ 20–40%), as well as in the left ventricular function GWAS studies were obtained based on UK Biobank individuals, who are also included in our exposure GWAS for BMI and BP. We note there is some differential power among outcome datasets, including the negative control outcome, in our study. This is because some of the more common cardiovascular outcomes included have now been analysed in very large meta-analyses. A full breakdown of sample sizes in all datasets included is provided in Additional file 1: Table S1.

### Genetic instrument selection

BMI and BP GWAS summary statistics were obtained in GWAS-VCF [48] format from the OpenGWAS platform and were subsequently converted to the *TwoSampleMR* [49] package format using the “gwasvcf_to_TwoSampleMR” function from the *gwasglue* [50] R package. Genetic instruments for each exposure were identified based on the conventional genome-wide significance threshold (*p* value < 5 × 10^−8^). Independent instruments were clumped using “*ld_clump*” wrapper for plink ver 1.943 [51] from the *ieugwasr* R package [52] based on strict linkage disequilibrium (LD) parameters (*r*^2^ was < 0.001 within 10 Mbp in 1000 Genomes [53] European panel). Note that strict clumping parameters have been applied to ensure all SNPs incorporated in our MR analyses are valid independent instrumental variables [54]. We sometimes used SNP proxies showing high genetic correlation (*r*^2^ > 0.8) in the instances when the chosen SNP was missing in the outcome dataset.

### Assignment of genetic instruments to pathways: MendelVar

Having obtained 887 independent genetic IVs for BMI, 914 for diastolic BP, and 863 for systolic BP, we used MendelVar [19] to partition IVs into subsets enriched for Mendelian disease categories. Briefly, we used the MendelVar [19] pipeline to generate LD-based genomic intervals around each input SNP (delimited by most distant SNP with minimum *r*^2^ = 0.8 within 1 Mbp in either direction). Then, we checked for enrichment of phenotype ontology terms linked to Mendelian disease genes (defined as coding region with 1000 bp 5′ and 3′ flanking regions) present within the interval using the INRICH [55] software contained in the MendelVar platform. In total, the trait associated SNPs were queried against the MendelVar database comprising ~ 11,500 disease/gene relationships, of which there are ~ 6800 distinct disease labels [19].

### Assignment of genetic instruments to tissues: colocalization

We followed the method described in Leyden et al. [11] to assign SNPs to subsets with evidence for colocalization with an eQTL in at least one of the two chosen tissue types (kidney and vasculature). For BP traits, we used cis-eQTLs in kidney (NephQTL2 [56] tubulointerstitial *n* = 311 and glomerular *n* = 240) and arteries (GTEx 8 [57] aorta *n* = 387 and coronary *n* = 212); similar sample sizes across tissues should result in comparable power. We used intervals of ± 100 kbp centred on each exposure SNP for colocalization in the coloc R software [58], and a stringent posterior probability H4 (PP_H4_, hypothesis 4: shared causal variant between exposure GWAS and cis-eQTL dataset) threshold of 0.9 to partition SNPs into coloc-based tissues.

### Enrichment analysis within subsets

We used ToppFun from ToppGene Suite [59] and over-representation module in ConsensusPathDB [60] (both with default settings) to test for global enrichment of functional terms in gene subsets identified by MendelVar and coloc harnessing popular ontologies, such as GO [61], Reactome [62], and KEGG [63].

### One-sample Mendelian randomization analyses

Our methodology for one-sample MR analyses followed the protocol described [11] and utilized individual-level data from the UK Biobank [64] (Application number: 81499). This involved the creation of separate genetic risk scores (GRS) for each group of SNPs: all SNPs (i.e. all trait associated SNPs identified in the GWAS results) and each of the subsets of those SNPs identified by the pathway-/tissue-partitioning methods. Weighted GRS were calculated based on the number of effect alleles possessed by each individual and weighted by the respective SNP effect sizes obtained from the original GWAS. Detail of QC applied to the individual-level genotype data in the UKB has been described in full previously [65]. Briefly, the included comprised 334,398 unrelated people of European descent and was established after excluding participants with withdrawn consent, genetically related individuals (*n* = 79,450), or those who did not cluster with “white European” group (*n* = 23,669) based on K-means clustering (K = 4). Additional standard exclusions were applied comprising mis-matches in genetically determined and self-reported sex, putative sex chromosome aneuploidy, and outliers in heterozygosity (*n* = 849).

We first evaluated the overall exposure effects (for SBP, DBP, and BMI) in standard MR analyses employing either linear or logistic regression using *AER* [66] and *mass* [67] R packages. We analysed all available outcomes (Additional file 1: Table S2: AF, HF, CHD, MI, T2D, stroke, AMD, LVEDV, LVESV, LVEF, SV) and adjusted for variables such as age, sex, the leading 10 principal components, and a binary marker for genotype chips. A rank-based inverse normal transformation was applied to continuous cardiac outcomes (LVEDV, LVESV, LVEF, SV) before analysis. We carried out further univariable MR analyses using each pathway- or tissue-partitioned GRS as exposures in turn. Finally, we deployed multivariable models which incorporated either the two pathway- or two tissue-partitioned GRS to quantify the direct effect of each subset on the outcome.

To estimate the type 1 error rate due to the fraction of shared individuals between our GRS construction GWAS datasets and the cases and controls in the UK Biobank sample, we used the “sample overlap” web app [47]. The results indicated that, based on the strong *F*-statistics of our instruments, the overlap of samples is unlikely to cause significant bias in our analyses (type 1 error rate < 0.05).

### Sensitivity analyses

We also used GWAS exposure and outcome datasets described above in two-sample MR analysis. Firstly, we carried out univariable MR analysis, using all top SNPs for a given exposure and then pathway or tissue subsets separately. Secondly, for tissue-partitioned subsets, we ran multivariable MR including both tissue subsets to obtain mutually adjusted “independent” effects as per [12]. This method is based on the application of multivariable MR whereby estimates on each exposure are obtained for all genetic instruments in the model [68]. It does so by taking advantage of the differential evidence for colocalization between tissues to differentially weight each instrument’s effect size by coloc’s PP_H4_ in each tissue-partitioned exposure, respectively. We were not able to carry out two-sample multivariable MR for pathway-partitioned SNPs due to lack of a suitable metric by which to scale individual variants’ effects. We chose the “TwoSampleMR” [49] R package for the standard inverse-variance weighted, mode-weighted, median-weighted and MR-Egger MR analyses as well as multivariable MR analysis. We then calculated instrument strength (*F*-statistics, *R*^2^) and heterogeneity (Cochran’s *Q* and *I*^2^) [69]. Steiger directionality tests were additionally performed on all partitioned exposure-outcome combinations examined which confirmed that our partitioned IVs do not produce estimates which are consistent with reverse cause.

In addition to including a negative control for the outcome (AMD), we also attempted to provide a negative control SNP partitioning for pathway-partitioned SNPs. To achieve this, we subset exposure SNPs by a feature which was not expected to biologically influence the outcome (mode of inheritance: autosomal dominant or recessive) and which was not significantly enriched in any blood pressure pathway partition. We did not run this control example for BMI, since we did find enrichment of autosomal dominant disease-assigned SNPs among mental health SNPs in the dataset, mostly driven by intellectual development disorders (χ^2^ = 10.3, *N* = 71, *p* value = 0.001, Supplementary Table 3).

### Random sampling of SNP subsets

Finally, we decided to empirically determine how often the difference in MR estimates between randomly drawn SNP subsets equals or exceeds the one observed for pathway- or tissue-partitioned instruments. When simulating subsets for comparison with pathway-partitioned instruments, we drew random *n*_1_ SNPs without replacement and then separately *n*_2_ SNPs without replacement (where *n*_1_ and *n*_2_ correspond to the number of SNPs in the original SNP subsets) to represent two SNP subsets which can randomly overlap. For tissue partitions, the procedure for drawing SNPs was modified to randomize the association between SNP, tissue, and colocalization probability. In that case, we first randomly permuted all PP_H4_ values across both tissues and variants, following which we extracted SNPs with PP_H4_ values above the chosen threshold (0.9 in our analysis and 0.8 in replication of Leyden et al. [11], see below) to be used as two random SNP subsets. We then ran equivalent 1-sample and 2-sample MR analyses as for the “true” pathway- and tissue-partitioned IVs. The entire procedure was repeated 1000 times per exposure-outcome combination and analysis type.

### *Replication of *Leyden et al. [11]

We re-analysed the Leyden et al. [11] dataset for select outcomes to compare the MR results using BMI exposures assigned by gene expression to the adipose (86 SNPs) and brain (140 SNPs) tissues with our pathway-partitioned approach. We also established the extent to which the difference between tissue-partitioned IVs could emerge by chance, using random instrument re-sampling as described above.

## Results

### Assignment of variants to pathways—MendelVar

To assign blood pressure SNPs to pathways, we leveraged data from the MendelVar resource. Using MendelVar, we assessed whether Mendelian disease genes (and their symptoms) were overrepresented among blood pressure GWAS loci or in strong LD (Fig. 1). The two most enriched terms were “abnormal renal morphology” (DBP: 90 SNPs, *p* value = 8 × 10^−5^; SBP: 84 SNPs, *p* value = 2.8 × 10^−4^, Additional file 1: Tables S4–S7) and “abnormal blood vessel morphology” (DBP: 79 SNPs, *p* value = 1.2 × 10^−4^; SBP: 83 SNPs, *p* value = 2 × 10^−5^) from the Human Phenotype Ontology (HPO) [70]. A proportion of SNPs in the “renal” and “vessel” subsets overlapped by ~ 28% (DBP, 37 SNPs) and ~ 33% (SBP, 42 SNPs) (Additional file 2: Fig. S1 A–B).

We then used independent ontologies not related to Mendelian disease (featured in ConsensusPathDB [60] and ToppGen [59]) to check if they provided orthogonal evidence for enrichment in a given pathway or tissue (Additional file 3: Supplementary tables enrichment (STE)). We found that kidney-related terms were significantly enriched in the “renal” gene set (Additional file 3: Supplementary Dataset STE 1–4), e.g. renal system development (Gene Ontology [71], DBP *q* value: 2.6 × 10^−9^; SBP *q* value: 4.7 × 10^−6^). We also observed significant overrepresentation of gene sets related to metabolism, hormonal regulation, type 2 diabetes, and cancer. Our “vessel” gene set was found to contain a strong overrepresentation of cardiovascular terms across many ontologies (Additional file 3: Supplementary Dataset STE 5–8): e.g. blood vessel development (Gene Ontology, DBP *q* value: 7.7 × 10^−6^; SBP *q* value: 4.8 × 10^−10^). To a lesser extent, we found enrichment of kidney-related terms (*renin secretion*, *EPO signalling pathway*) in the “vessel” gene set and cardiovascular-related terms (*blood vessel development*, *heart development*) in the “renal” set which is not unexpected given the overlap of “renal” and “vessel” SNPs in our partitions.

For BMI, we used the Alliance of Genome Resources slim version of Disease Ontology [72] (28 general disease types). The top most enriched categories were “disease of metabolism” (45 SNPs, *p* value = 8.8 × 10^−4^, Supplementary Tables 8–9) and “disease of mental health”/“developmental disorder of mental health” (39 SNPs, *p* value = 4.1 × 10^−3^). Six SNPs (~ 7%) were shared between the “metabolic” and “mental health” sets (Supplementary Fig. 1 C). The evaluation of “mental health” gene sets for enrichment of non-Mendelian disease functional ontologies highlighted neuronal mechanisms, in particular synaptic signalling, e.g. neuronal system (Reactome [73], *q* value: 1 × 10^−3^). The strongest enrichment of terms in the “metabolic” gene set were related to metabolism and type 2 diabetes, e.g. metabolism (Reactome, *q* value = 5 × 10^−11^). Further details provided in Additional file 3: Supplementary Dataset STE 9–12.

### Assignment of variants to tissues—coloc

To stratify blood pressure SNPs by SNPs which primarily effect gene expression in the kidney or vasculature, we applied the previously proposed stratification approach [11] which is based on evidence for colocalization with gene expression, to assign SNPs to tissues (Additional file 1: Tables S10–S11). We applied a high minimum threshold posterior probability of colocalization (PP_H4_ > 0.9) to assigns SNPs to either (1) the aorta and coronary tissues (referred to collectively as “artery”) or (2) the glomerular or tubulointerstitial tissues (referred to collectively as “nephro”). Using this criteria, 117 SNPs were assigned to “artery” tissue for DBP and 132 for SBP. Among “artery” SNPs, most loci colocalized in the aorta (108 for SBP, 126 for DBP; Additional file 2: Fig. S1D–E). Eighty-seven and 77 SNPs were assigned to the “nephro” tissue group for DBP and SBP, respectively (Additional file 1: Tables S6–S7). Among “nephro” SNPs, tubulointerstitial tissue dominated with 65 and 64 SNPs for DBP and SBP (Additional file 2: Fig. S1D–E). Approximately 25% and 20% of SNPs were common to both tubulointerstitial and glomerular in the DBP and SBP sets, respectively. Comparison of all “artery” and “nephro” SNP sets revealed approximately 21% (36 SNPs) and 25% (42 SNPs) overlap for DBP and SBP, respectively.

Tissue-partitioning showed limited alignment with functional pathways relevant to each tissue (Additional file 3: Supplementary Dataset STE 13–20), with very few weakly enriched terms found overall: 4 for “artery” SNPs in DBP (hypertrophic cardiomyopathy, ACE inhibitor pathway, metabolism of lipids, mitochondrial electron transport chain; *q* value = 0.029–0.047), 3 for “nephro” SNPs in DBP (O-glycosylation of TSR domain-containing proteins, aquaporin-mediated transport, transport of small molecules; *q* value = 0.024–0.047), and 3 cardiomyopathy terms were enriched for among “nephro” SBP genes (*q* value = 0.017–0.024).

BMI colocalization results were obtained from Leyden et al. [11]. Among those, 140 SNPs were assigned to the brain and 86 to the adipose tissue, with 43 overlapping (~ 23% of total set). Enrichment of genes with colocalization evidence for BMI in adipose and brain tissue sets showed limited overlap with biologically relevant terms, especially for the brain (Additional file 3: Supplementary Dataset STE 21–24); these replicated analyses previously described [11]. Detail of all trait associated genetic instruments and their pathway and tissue partition annotations are provided in Additional file 1: Tables S6, S7, and S9.

### Comparison of pathway- and tissue-partitioned SNP sets

Importantly, comparison of the SNP sets assigned using pathway- and tissue-partitioning showed that they are largely distinct and could potentially offer orthogonal evidence (Additional file 2: Fig. S1 F–G). Only ~ 14% of SNPs were assigned to a category by both methods: 33 DBP and 34 SBP SNPs compared to 234 and 234 uniquely assigned SNPs, respectively. Similar observations were made for BMI (Additional file 2: Fig. S1H–I): only 5 out of the 45 “mental health” pathway SNPs were shared with tissue SNPs as assigned previously by Leyden et al. [11]—4 with “brain” and 1 with both “adipose”/“brain”. A larger overlap was observed for “metabolic” pathway SNPs: 17 out of 39 SNPs were shared with tissue subsets (3 with “adipose”, 5 with both “adipose”/“brain”, and 9 with “brain”).

### Pathway- and tissue-partitioned Mendelian randomization analyses for blood pressure

Having established the pathway- and tissue-partitioned SNPs for blood pressure, we proceeded to use them as exposures in one-sample MR carried out in individuals of European ancestry in the UK Biobank. We focussed on selected cardiometabolic outcomes (Additional file 1: Table S2), as they have been firmly established to be causally influenced by blood pressure [25, 25, 74, 75] and were previously evaluated using the tissue-partitioning approach with respect to BMI by Leyden et al. [11]. We describe in further detail the results for CHD, SV, and T2D below.

### Pathway- and tissue-partitioned Mendelian randomization analyses of CHD

**Fig. 2:**
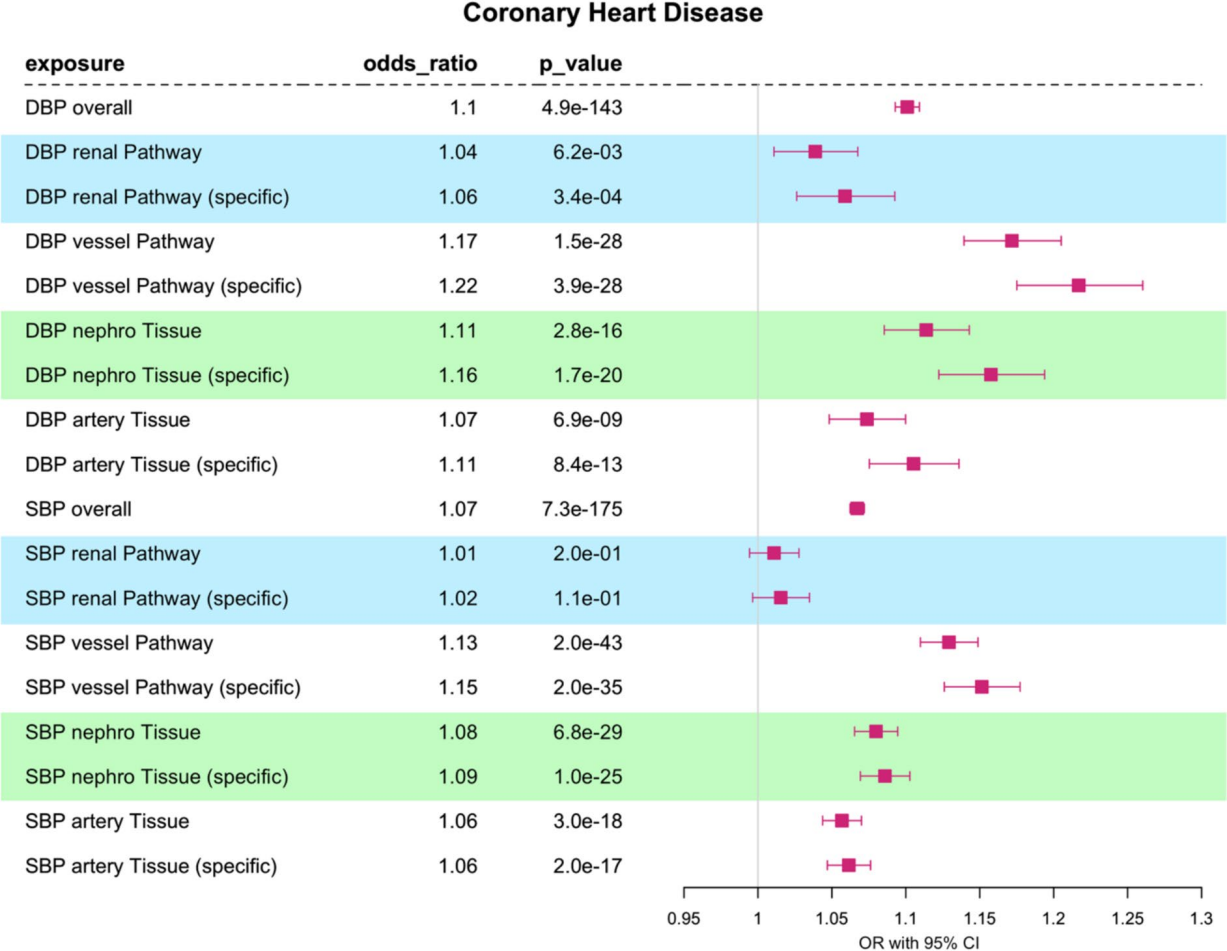
Coronary heart disease: one-sample multivariable Mendelian randomization analysis of the effect of diastolic blood pressure (DBP) and systolic blood pressure (SBP) on CHD. We have investigated the overall trait effect using univariable MR and have conducted multivariable analyses of pathway-partitioned instruments (informed by Mendelian disease with abnormalities in the *renal* or blood *vessel* system) and multivariable analyses of tissue-partitioned genetic instruments (informed by evidence for genetic colocalization with gene expression “*nephro*” (kidney tissues: glomerular and tubulointerstitial) and “*artery*” (aorta and coronary artery tissues) instruments). Effect sizes are scaled to per one SD change in blood pressure

We confirmed that genetically predicted DBP and SBP have an overall causal relationship with coronary heart disease (CHD) when instrumented using all trait associated IVs (OR_95%CI_ = 1.09–1.11 and OR_95%CI_ = 1.06–1.07, respectively) (further details Additional file 1: Table S12). We next conducted pathway-partitioned MR by incorporating the “renal” and “vessel” assigned pathway IVs and adjusted for shared effects in a multivariable setting. Our results indicate that the positive-directional effect of DBP and SBP on CHD is driven by the “vessel” pathway (DBP: OR = 1.17, OR_95%CI_ = 1.14–1.21, *p* value = 1.5 × 10^−28^; SBP: OR = 1.13, OR_95%CI_ = 1.11–1.15, *p* value = 2 × 10^−43^), relative to the “renal” pathway (DBP: OR = 1.04, OR_95%CI_ = 1.01–1.07, *p* value = 6.2 × 10^−3^; SBP: OR = 1.01, OR_95%CI_ = 0.99–1.02, *p* value = 1.9 × 10^−1^). 95% confidence intervals of the two subsets are distinct and non-overlapping (Fig. 2, Additional file 1: Table S13).

Interestingly, the results of our tissue-partitioned MR analysis incorporating “nephro” and “artery” SNPs assigned by evidence for colocalization yielded a distinct trend (Additional file 1: Table S14). In this analysis, stronger positive directional effects were observed for the “nephro” tissue assigned IVs on CHD (DBP: OR = 1.11, OR_95%CI_ = 1.08–1.14, *p* value = 2.8 × 10^−16^; SBP: OR = 1.08, OR_95%CI_ = 1.06–1.09, *p* value = 6.8 × 10^−29^) when evaluated against “artery” tissue in the MVMR model (DBP: OR = 1.07, OR_95%CI_ = 1.05–1.10, *p* value = 6.8 × 10^−9^; SBP: OR = 1.06, OR_95%CI_ = 1.04–1.07, *p* value = 3 × 10^−18^). All tissue-partitioned exposures maintained evidence of an effect on CHD for SBP and DBP, unlike “renal” pathway-partitioned SBP which did not maintain evidence for an effect on CHD (*p* value = 0.19).

A sensitivity analysis limiting exposures to SNPs specifically assigned to each subset resulted in similar estimates as when using all pathway/tissue assigned SNPs. We also show similar results for myocardial infarction (Additional file 2: Fig. S2) which is closely genetically correlated to CHD (Additional file 2: Fig. S3). Point estimates for MI display greater uncertainty and while the difference between pathway-partitioned exposures persists, it was not apparent for “artery” and “nephro” tissue partitions.

### Pathway- and tissue-partitioned Mendelian randomization analyses of SV

**Fig. 3:**
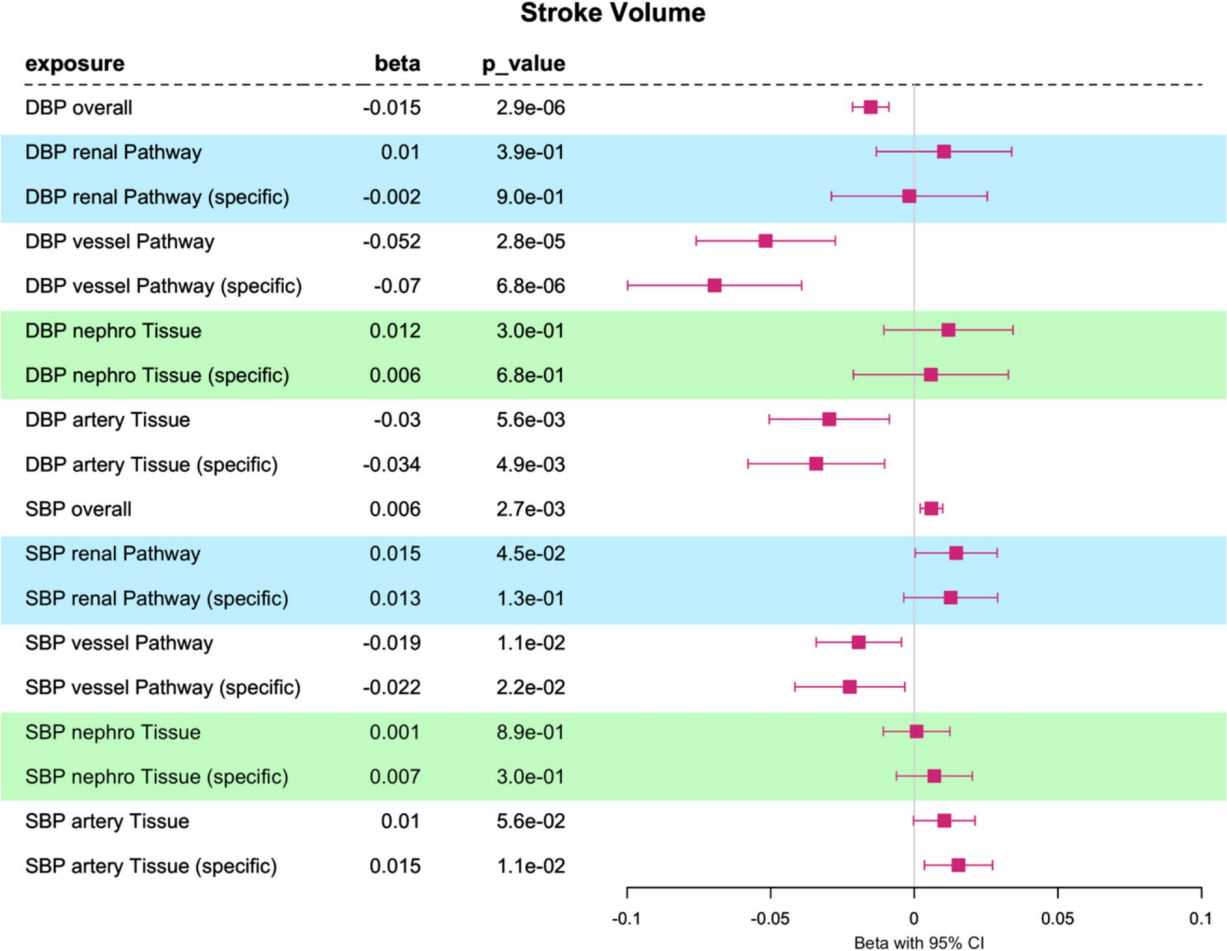
Stroke volume: one-sample multivariable Mendelian randomization analysis of the effect of diastolic blood pressure (DBP) and systolic blood pressure (SBP) on stroke volume (SV). We have investigated the overall trait effect using univariable MR and have conducted multivariable analyses of pathway-partitioned instruments (informed by Mendelian disease with abnormalities in the *renal* or blood *vessel* system) and multivariable analyses of tissue-partitioned genetic instruments (informed by evidence for genetic colocalization with gene expression “*nephro*” (kidney tissues: glomerular and tubulointerstitial) and “*artery*” (aorta and coronary artery tissues) instruments). Effect sizes are scaled to per one SD change in blood pressure

The pathway- and tissue-partitioned IVs showed consistent directional effects for left ventricular stroke volume (SV, Fig. 3). Analysis of the overall trait effect yields a negative directional effect on SV for genetically predicted DBP using all non-stratified instruments (beta = − 0.015, beta_95%CI_ = (− 0.021, − 0.008), *p* value = 2.9 × 10^−6^). Both “vessel” (beta = − 0.052, beta_95%CI_ = (− 0.076, − 0.028), *p* value = 2.8 × 10^−5^) and “artery” instrumented DBP (beta = − 0.03, beta_95%CI_ = (− 0.051, − 0.009), *p* value = 5.6 × 10^−3^) maintained evidence for the negative directional effect of DBP on SV, while a “renal” (beta_95%CI_ = − 0.013, 0.034) or “nephro” (beta_95%CI_ = − 0.011, 0.034) DBP effect was not supported. The opposite was observed for SBP, which yielded an overall positive directional effect on SV using all non-stratified instruments (beta = 0.006, beta_95%CI_ = (0.002, 0.01), *p* value = 2.7 × 10^−3^). Our pathway-stratified MR indicated that the “renal” pathway SNPs contribute to the overall positive directional effect of SBP (beta_95%CI_ = (0.0003, 0.0289)) while “vessel” SNPs have a negative directional effect on SV (beta_95%CI_ = (− 0.034, − 0.004)). No clear distinction for tissue-partitioned exposures was observed on SV.

### Pathway- and tissue-partitioned Mendelian randomization analyses of T2D

**Fig. 4:**
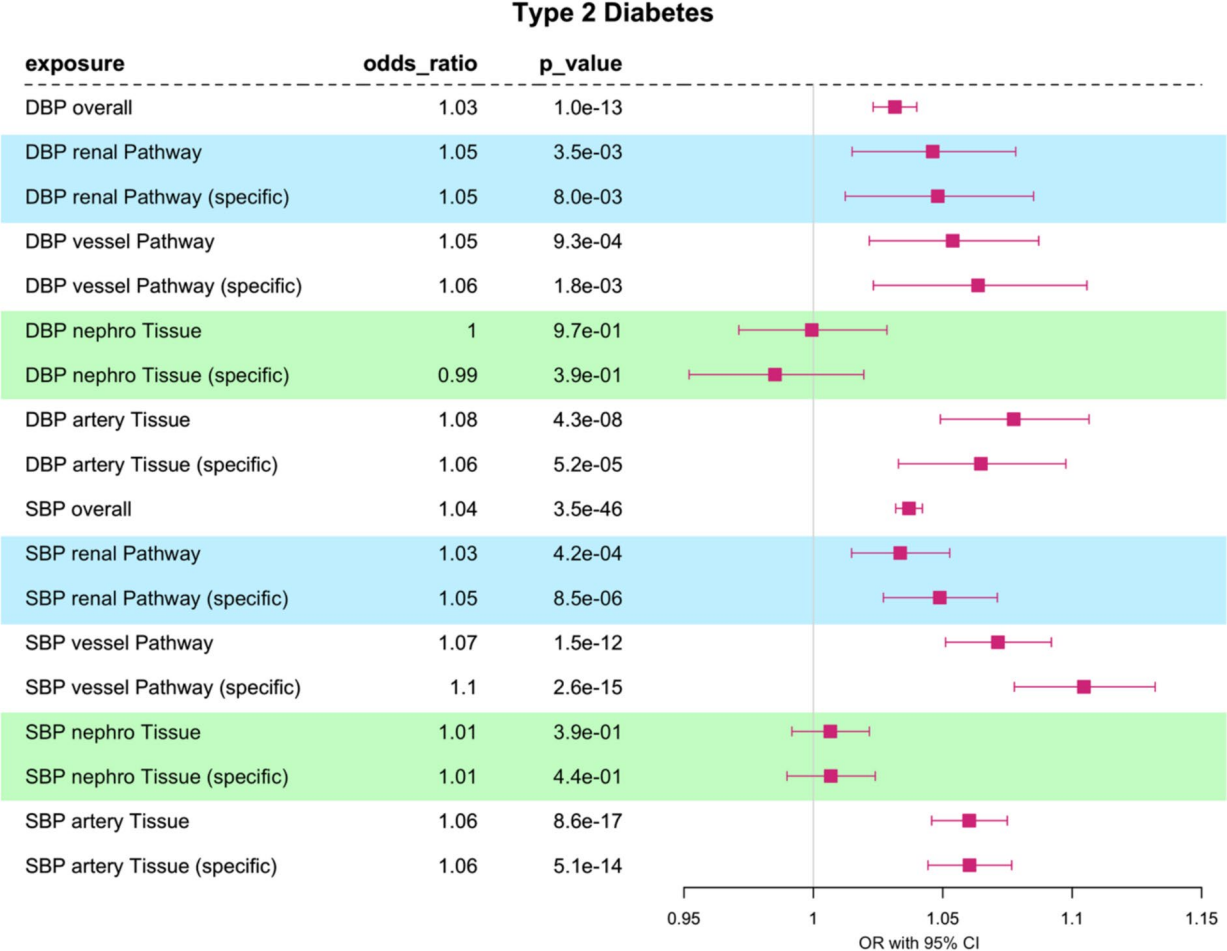
Type 2 diabetes: one-sample multivariable Mendelian randomization analysis of the effect of diastolic blood pressure (DBP) and systolic blood pressure (SBP) on T2D. We have investigated the overall trait effect using univariable MR and have conducted multivariable analyses of pathway-partitioned instruments (informed by Mendelian disease with abnormalities in the *renal* or blood *vessel* system) and multivariable analyses of tissue-partitioned genetic instruments (informed by evidence for genetic colocalization with gene expression “*nephro*” (kidney tissues: glomerular and tubulointerstitial) and “*artery*” (aorta and coronary artery tissues) instruments). Effect sizes are scaled to per one SD change in blood pressure

Dissecting the pathway and tissue effects on type 2 diabetes also reveal a heterogenous landscape (Fig. 4). We first confirmed the known positive directional effect of blood pressure captured by the overall effect on T2D (DBP OR_95%CI_ = 1.02–1.04; SBP OR_95%CI_ = 1.03–1.04). Our pathway-stratified estimates for DBP maintained comparable positive directional effects on the risk of T2D. While the “vessel” SBP exposure produced higher odds of T2D (OR = 1.07, OR_95%CI_ = 1.05–1.09, *p* value = 1.5 × 10^−12^) than “renal” (OR = 1.03, OR_95%CI_ = 1.01–1.05, *p* value = 4.2 × 10^−4^), we note that the effect estimates are partially overlapping. Further, the results of our tissue-partitioned MR for “artery” maintains a positive directional effect on the risk of T2D for both DBP (OR = 1.08, OR_95%CI_ = 1.05–1.11, *p* value = 4.3 × 10^−8^) and SBP (OR = 1.06, OR_95%CI_ = 1.04–1.07, *p* value = 8.6 × 10^−17^), while null effects were observed for “nephro” SNPs (DBP OR_95%CI_ = 0.97–1.03; SBP OR_95%CI_ = 0.99–1.02).

We repeated the analysis above by analysing each partitioned exposure independently within an individual-level univariable MR framework (Additional file 1: Tables S13–S14). The results of this analysis provided similar patterns between SNP subsets, with any differences in effect estimates accentuated further in MVMR analyses.

### Sensitivity analyses for blood pressure

We further evaluated the exposure-outcome relationships in a summary-level “two-sample” MR setting (Additional file 1: Tables S15–S17). In general, the two-sample analyses had less power to detect distinct effects between partitioned exposures, but were directionally consistent with the individual-level (“one-sample”) MR. The one exception was a more pronounced causal effect of “nephro” SBP on myocardial infarction (OR = 1.04, OR_95%CI_ = 1.02–1.06, *p* value = 2.3 × 10^−4^) relative to “artery” (OR = 1, OR_95%CI_ = 0.98–1.02, *p* value = 1), aligning with the dominant role of “nephro” SBP observed in our one-sample MVMR of the related outcome CHD.

We found that mean absolute effect sizes tend not to differ significantly between all SNPs and pathway-/tissue-partitioned IVs (Supplementary Table 20). Among all partitioned exposures, the mean *F*-statistics were > 60, suggesting low risk of weak instrument bias.

We proceeded to evaluate changes in the average heterogeneity (*Q*_het_ = *Q*/(*Q*_df_ − 1)) within pathway- or tissue-partitioned IVs in comparison to all trait-associated instruments. We did not detect a downward trend in average heterogeneity which instead varied in unexpected ways. For example, we found *Q*_het_ to be lower among pathway-partitioned and tissue-partitioned exposures relative to all DBP SNPs (Additional file 1: Tables S21–S23). However, for SBP “renal” pathway and “artery” tissue IVs showed higher *Q*_het_ than all SBP SNPs, while “vessel” pathway and “nephro” tissue IVs were the opposite.

Next, we repeated our analyses using a negative outcome phenotype: age-related macular degeneration [76] (AMD). The one-sample and two-sample MR results (Additional file 1: Tables S13–S14, S16–S17) did not indicate strong evidence of causal effect for our pathway- or tissue-partitioned exposures, in line with our expectations. We did find one weak non-null result, however, only at a nominal level (Supplementary Fig. 4) for SNPs specific to the “renal” pathway for DBP and SBP (DBP OR = 1.07, OR_95%CI_ = 1–1.14, *p* value = 0.03; SBP OR = 1.04, OR_95%CI_ = 1.00–1.08, *p* value = 0.03).

Negative control partitioning of exposures by the main modes of Mendelian disease inheritance (autosomal “dominant” or “recessive”) showed no association with the pathway-partitioned “renal”- “vessel” subdivision (Supplementary Fig. 5 A, B) for DBP (*p* value = 0.13) and SBP (*p* value = 0.4, Supplementary Table 3). We note that this sensitivity is based on the assumption that the negative control feature is not associated with pathway or tissue in a biologically meaningful way, though this may not hold in all cases. For example, a difference between “dominant” and “recessive” SNPs was detected when focussing on CHD as the outcome in a one-sample MR analysis (Supplementary Fig. 6). For instance, all “dominant” DBP SNPs had a higher effect on CHD (OR = 1.12, OR_95%CI_ = 1.09–1.14, *p* value = 3.9 × 10^−23^) than all “recessive” SNPs (OR = 1.08, OR_95%CI_ = 1.05–1.10, *p* value = 1.8 × 10^−11^) (Additional file 1: Table S22). However, when limiting ourselves to SNPs “specific” to each subset, the opposite conclusion was obtained with higher effects seen for “recessive” (OR = 1.22, OR_95%CI_ = 1.17–1.26, *p* value = 3.9 × 10^−28^) than “dominant” SNPs (OR = 1.06, OR_95%CI_ = 1.03–1.09, *p* value = 3.4 × 10^−4^). No meaningful difference between the SNP subsets was observed in two-sample MR results (Additional file 1: Tables S23, S24). In addition, much less variation across SNP subsets was found for the T2D outcome (Additional file 2: Fig. S7), apart from one outlier: specific “recessive” SBP.

### Pathway- and tissue-partitioned Mendelian randomization analyses for body mass index

We conducted a further comparison of the pathway- and tissue-partitioned MR methods for BMI. The largest differential effect between pathway-partitioned BMI was observed between “mental health” and “metabolic” SNPs on atrial fibrillation. The overall trait effect predicted using all non-stratified BMI SNPs confirmed a moderate effect on AF (OR = 1.05, OR_95%CI_ = 1.04–1.06, *p* value = 4.6 × 10^−55^; Additional file 1: Table S25). Our individual-level MVMR analysis highlighted this effect was maintained predominantly by the metabolic pathway (OR = 1.10, OR_95%CI_ = 1.07–1.13, *p* value = 3.3 × 10^−10^) when adjusting for “mental health” (OR = 1.05, OR_95%CI_ = 1.02 = 1.08, *p* value = 5.6 × 10^−4^) in the same model (Additional file 1: Table S26). A similar differential relationship was captured between “all”, “metabolic”, and “mental health” pathways in 1-sample univariable analyses (Additional file 1: Table S26) and 2-sample MR analyses (Additional file 1: Tables S27–S28). This provides orthogonal evidence to the tissue-partitioned MR result reported previously [11] and replicated here (Additional file 1: Tables S29–S32), where the “brain” IVs predominantly maintained the positive directional effect on AF (1-sample MVMR OR = 1.04, OR_95%CI_ = 1.02–1.06, *p* value = 6.4 × 10^−6^, Additional file 1: Table S30), compared to “subcutaneous adipose” (1-sample MVMR OR = 1.02, OR_95%CI_ = 1–1.04, *p* value = 0.08).

### Sensitivity analyses for body mass index

The average heterogeneity (*Q*_het_) of “mental health” and “metabolic” BMI SNPs for AF was close to the average heterogeneity for all BMI SNPs (Additional file 1: Tables S33–S34)—2.5, 2.05, and 2.19, respectively. We also did not uncover evidence for a systematic reduction in average heterogeneity among SNP subsets across other outcomes.

The results from our negative control outcome analysis of AMD indicated that “mental health” and “metabolic” SNP subsets analysed independently in 1-sample/2-sample univariable MR or in conjunction with each other (1-sample multivariable MR) show no evidence for causal role, as per expectations (Additional file 1: Tables S26, S28).

### Random sampling of SNP subsets

Overall, the results of our one-sample pathway-partitioned MVMR analysis yielded a variety of differential effect relationships depending on the outcome. These ranged from negligible (e.g. DBP Mendelian partitioning with respect to T2D), medium with overlapping confidence intervals (SBP Mendelian partitioning with respect to T2D) to totally distinct (SBP Mendelian partitioning with respect to CHD). Where negligible differences are observed between stratified exposure effects on an outcome in this context, we would interpret this as neither pathway explaining more of the trait effect on the outcome than the other. Where totally distinct effects are observed this provides evidence to support a direct effect on the outcome by one pathway independent of the other. Furthermore, our results yielded examples where there was alignment between the pathway and tissue underlying the exposure-outcome relationship identified by both the pathway- and tissue-partitioning methods (e.g. vessel- and artery-assigned SBP instruments produced greater effect sizes on T2D than renal- and nephro-assigned instruments), and where distinct exposure-outcome effects were estimated between methods (e.g. vessel-assigned instruments and nephro-assigned instruments for SBP each had greater effects than renal- or artery-assigned instruments respectively on CHD). The latter case emphasizes intriguing differences inherent to the SNPs characteristics captured by each stratification method, i.e. symptomatic effects on the biological system (pathway-partitioning) or gene expression effects in the tissue of origin (tissue-partitioning). Therefore, it is important to establish whether any differences detected were likely to be both biologically meaningful and not driven by chance assortment.

To address this, we employed a simulation technique, where we re-ran our analysis pipeline 1000 times for each exposure-outcome pair using 2 subsets of SNPs sampled randomly from across all the exposure SNPs. This allowed us to quantify the frequency of the absolute differences in effect sizes such as observed in our MR analysis (or greater) relative to background (in the process obtaining two-tailed *p* value with the floor value of 10^−3^), and empirically derive 95% confidence intervals of the difference (Additional file 1: Table S35).

**Fig. 5:**
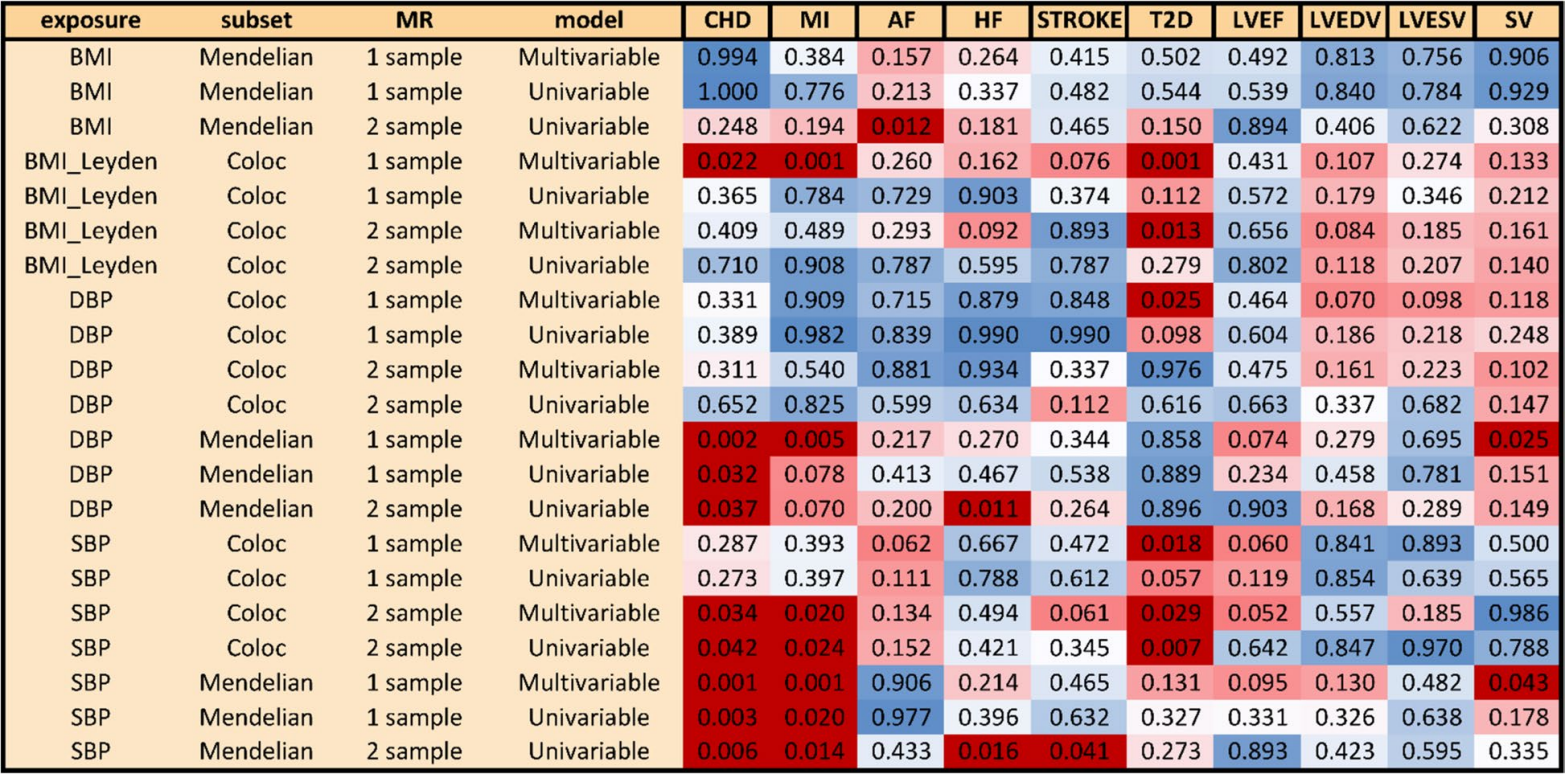
Matrix of empirically derived (1000 replicates) *p* values for distribution of effect size differences between pathway-partitioned (Mendelian) instruments and tissue-partitioned (coloc) instruments in 1-sample and 2-sample MR setting (univariable and multivariable) using body mass index (BMI) and blood pressure—systolic (SBP) and diastolic (DBP) as exposures and cardiometabolic traits as outcomes (AF, CHD, MI, HF, stroke, T2D, LDVEDV, LVEF, LVESV, SV). *p* values < 0.05 are highlighted in dark red

Figure 5 provides an overview of this sensitivity approach run for all the exposures (BMI, SBP, DBP), SNP subsets (pathway, tissue), MR methods (1-sample or 2-sample), and MR models (univariable, multivariable). Across all the MR analyses with blood pressure as exposure, we find strong evidence for the differential effect of pathway-assigned “renal” and “vessel” exposures on CHD (*p* value range: 0.001–0.042) along with MI. In addition, tissue-assigned “artery” and “nephro” SBP subsets show a strong difference of effect on T2D, CHD, and MI (*p* value range: 0.007–0.042) but the latter two only in the two-sample MR setting. Significant evidence based on 1-sample MVMR was found among tissue-partitioned SNP subsets for DBP and T2D, as well as BMI and CHD/MI. Both 1-sample and 2-sample MVMR analyses support a differential effect of tissue-partitioned “adipose” and “brain” SNPs on BMI (*p* value = 0.001–0.013).

We also find weaker evidence for a difference between “renal” and “vessel” blood pressure subsets with respect to the stroke volume outcome (1-sample MVMR SBP *p* value = 0.043, DBP *p* value = 0.025). There was little evidence against the null hypothesis (of chance SNP assortment) for any of the BMI or BP pathway-based results with respect to atrial fibrillation, heart failure, stroke, LVEDV, LVESV, or LVEF, with the exception of the following two-sample univariable MR analyses involving pathway-partitioned instruments: BMI effect on atrial fibrillation (*p* value = 0.012), systolic and diastolic BP effect on heart failure (*p* value < 0.02).

## Discussion

In this study, we have leveraged biological data to dissect the causal associations between blood pressure and BMI with cardiometabolic traits within an MR framework. To do so, we stratified trait associated genetic instruments by “pathway” (based on Mendelian disease categories using MendelVar) or by “tissue” (based on genetic colocalization with tissue derived eQTL).

A primary aim of the partitioning methods described is to assign a molecular context for how genetic IVs contribute to variation of a complex exposure. The identification of distinct estimates in MR analyses of partitioned exposures could arise from (1) differential pathway effects due to the measured exposure comprising phenotypic composites, or (2) no true differential pathway but differences emerging due to horizontal pleiotropy. In our application, the interpretation of results aligns with the former. Chiefly, BMI is a crude composite measure of a set of underlying traits. For example, measured BMI is downstream of adiposity, which is itself a complex trait comprising multiple adipose depots with differing consequences on disease [77, 78]. Similarly, this logic is extended to consider biological influences on measured blood-pressure (representing the measurable output from a highly dynamic homeostatic system, influenced by multiple organ-specific and centrally regulated biological mechanisms). The derivation of pathway- and tissue-partitioned exposures leverages biological data to directly address whether the overall exposure effect on the outcome is differentially influenced via a particular pathway/tissue “partition”. Importantly, we advocate that applications of the pathway- or tissue-partitioning methods should be accompanied by characterization of the IV partitions with respect to distinct trait effects where possible. This was demonstrated here first by cross-evaluation of enriched functional terms in independent data-resources, and secondly in a stringent sensitivity test against 1000 randomly sampled partition combinations. We note also that the “brain” and “adipose”-BMI IVs have been further shown to differentially correlate with distinct adiposity and anthropometry measures [11]. This is because interpretation of differential pathway/tissue-assigned MR estimates is informed by our confidence that the instrument-stratification procedure has appropriately identified distinct biology underlying the measured exposure.

Overall, our results highlight the significance of renal and vascular pathways in blood pressure-related conditions. Notably, vessel-pathway effects and kidney-tissue effects were each emphasized by the pathway- and tissue-stratified MVMR analyses respectively for SBP and DBP on CHD. Similarly for BMI, for example, we observed effects on atrial fibrillation which were pathway (“metabolic”-led) and tissue (“brain”-led). Sensitivity analyses emphasized the consistent direction of effects in one-sample and two-sample MR (univariable and multivariable) albeit with some variability in magnitude. We also conducted simulations to assess the probability that differences in causal estimates between SNP subsets arose by chance, and these support the interpretation of our main findings. In our main results, we have presented representative cardiac traits—CHD and SV, which as shown in Fig. S2 are highly genetically correlated with MI and LVEDV/LVESV, respectively. As expected, and demonstrated in Fig. 5, magnitude of effect size differences between pathway- and tissue-partitioned SNP subsets for those related exposures is highly congruent.

Where feasible, we aimed to repeat all multivariable analyses in both an individual (“one-sample”) and summary (“two-sample”) setting. Altogether, we find a comparable number of results [14] with empirically determined significant differences (*p* value < 0.05) in one-sample (univariable: 3, multivariable: 11) versus two-sample MR (univariable: 10, multivariable: 4), with univariable and multivariable results agreeing in the direction and magnitude of effect for the same SNP subsets. Some results reassuringly aligned across all MR types and model specifications: e.g. pathway-stratified analyses for DBP versus CHD, tissue-stratified analyses for SBP and BMI versus T2D. In some cases, pronounced differences were only observed in a single setting using one model type but not the others, such as tissue-partitioned analyses for BMI with respect to CHD (1-sample multivariable). In this scenario, we note that power to detect independent effects in the tissue-partitioned 2-sample MVMR model may be more of a challenge due to greater noise in the model. Despite the tissue-partitioned IVs being shown not to suffer from weak instrument bias previously [12], the incorporation of the differentially weighted versions of the same genetic “exposure” will have less precision. Related to this, the multivariable analyses in the present study restricted all pathway/tissue comparisons to two categories. This is because the MVMR is dependent on the power to detect strong trait effects which persist after adjusting for the other exposure in the same model. The identification of appropriate resources to expand this methodology will be an interesting area for future research. As such, weighing the balance between being able to identify distinct biological mechanisms and retaining sufficient power in the model is an important aspect of the multivariable study design we have applied here.

Therefore, to gain a better understanding of tissue- or pathway-partitioned effects, multiple types of MR analyses should be undertaken, whenever possible. In the present study, for ventricular function traits which were only available in the UKB cohort, we decided to focus on the individual- over the summary-level analysis. This is because the two-sample MR method may be more prone to over-fitting in the presence of high sample overlap of UKB individuals with our exposure sample. Furthermore, the current pathway-stratified MR approach is limited in that we are unable to model the SNP subsets jointly using the multivariable approach in a two-sample MR setting.

It may be expected that stratification of SNPs by pathway and tissue would result in reduced heterogeneity compared to using all SNPs, which can capture a wider range of biological processes. However, our pathway- and tissue-stratified results did not consistently support this expectation. For some IV subsets, the heterogeneity was indeed lower, which was in line with the initial hypothesis. However, other partitions showed greater heterogeneity than all SNPs combined, suggesting that those specific pathways or tissues might still be highly pleiotropic or potentially indicate the presence of some bias, such as misclassification of SNP functional category. While categorization of trait associated SNPs by their pathway or tissue helps bring us closer to understanding context-specific biological effects, there remains many levels of dynamic biological and molecular mechanisms which require further investigation. A recent example from Suzuki et al. highlights the potential for single-cell type omics platforms to inform partitioning of trait associated SNPs representing aetiological heterogeneity underlying T2D [79]. As the sample sizes of large complex trait GWAS grows, such as the most recently published blood-pressure meta-analysis [80], we may have even greater power to detect trait effects. With the advent of multi-omic and cell-specific QTL resources, it will be important to explore how these classifications may be refined in future.

Our new approach uses enrichment of ontology terms assigned to Mendelian diseases whose causal genes share the same genetic locus as the exposure SNPs, which can sometimes result in ambiguous assignment to appropriate biological pathways. For example, rs12630999 from BMI was allocated to both the “mental health” and “metabolic” set due to location between two neighbouring Mendelian disease genes: *STAG1* and *PCCB*, respectively. In another case, one systolic BP variant (rs3915499) was associated with two different pathways (“renal” and “vessel”) due to the two distinct monogenic diseases caused by disruption of smooth muscle myosin heavy-chain 11 (*MYH11*) gene [81] whose intron the SNP resides in. Under such a scenario, MendelVar cannot provide a more nuanced prediction regarding the “correct” pathway(s). In this example, “vessel”-only assignment could be more suitable as the associated disease, familial thoracic aortic aneurysm 4 is defined by profound structural abnormalities in the aorta, while a renal symptom (hydronephrosis) is only a marginal feature of megacystis-microcolon-intestinal hypoperistalsis syndrome 2. Nonetheless, we found that in many cases MendelVar unequivocally assigned SNPs to genes causal for disorders with strong links to a single category: “renal”—*CEP164* (nephronophthisis 15), *NRIP1* and *PBX1* (congenital anomalies of kidney and urinary tract syndromes); “vessel”—*EIF2 AK4* (familial pulmonary capillary hemangiomatosis), *PRDM6* (patent ductus arteriosus), *HTRA1* (cerebral arteriopathy with subcortical infarcts and leukoencephalopathy); “mental health”—*PPP3 CA* (developmental and epileptic encephalopathy 91, ACCIID), *KCNMA1* (cerebellar atrophy, developmental delay, and seizures, Liang-Wang syndrome); “metabolic”—*PPARG* (familial lipodystrophy), *SLC2 A2* (Fanconi-Bickel syndrome). Correct SNP assignment to the gene in each case was also supported by either presence within its intron or coding region.

A limitation of the pathway-stratified method is that it is reliant on mainly manual curation of disease ontology terms based on descriptions of clinical features [70, 72], and expanding the automation of that time-consuming process could increase power and potentially accuracy of our method. The specificity of the colocalization method suffers from co-ordinated expression of genes across many tissues which complicates selection of biologically causal tissues over merely tagging but new methods are being developed to address this confounding factor [82, 83]. We note that the modest eQTL sample sizes analysed in this study may have reduced the number of instruments we were able to identify with robust colocalization evidence [84]. However, as the catalogues of molecular trait data become more comprehensive, the role of single-cell type derived QTL in the identification of more specific instrument annotations will be a valuable area of future research. As the depth of available biological and molecular annotations develops, we may also achieve more precise IV partitioning with respect to biological function between pathway or tissue subsets. Furthermore, integrating different features to partition complex genetic exposures into a single joint model may offer improvement in pathway-based stratification. Here, we used Mendelian disease genetics and tissue derived gene expression, but other orthogonal evidence such as protein–protein interactions [85] and PheWAS [9] could be combined. Such (and other) future new methods offer a powerful means to advance our biological understanding of complex risk factor effects on disease and inform the development of targeted therapeutics.

Similarly to Darrous et al. [9], we conclude that our new pathway-stratified method offers complementary, orthogonal evidence to the existing tissue-stratified approach as evidenced by very weak overlap. Comparison of MR results using both approaches suggests agreement for the dominant role of vasculature-related SNPs in determining the left ventricular stroke volume and the risk of type 2 diabetes. Furthermore, the identification of a dominant role for blood pressure on CHD by “vessel” (via pathway-stratified MR) and “nephro” (via tissue-stratified MR) effects is a strength of the different biological characteristics captured by the data informing each approach. While gene selection based on coloc had less enrichment of relevant gene functional categories in general (Additional file 3: STE 21–24), the MendelVar selection by disease symptom ontology is a priori more directly related to gene function than expression pattern. We find more sharing of pathway-stratified “metabolic” than “mental health” SNPs with tissue-stratified “brain” SNPs, aligning with the brain-centric expression of key mechanisms regulating metabolism [86, 87], and the representation of brain regions relevant to homeostasis and energy balance in the brain eQTL data used for SNP identification [88]. Although both “metabolic” pathway and “adipose” tissue IVs are enriched for metabolic pathway genes, the “metabolism” term is broad and there could still be differences in the specific metabolic mechanisms represented in each subset.

## Conclusion

This study introduced and evaluated a novel, Mendelian disease-centric approach, “pathway-partitioned MR”, to dissect the impact of distinct biological pathways underlying complex risk factors on health outcomes. The comparison of results based on SNPs annotations using both the pathway- and tissue-partitioning methods demonstrates how integrating additional biological data can yield complementary insight on latent variables underlying complex genetic exposures. Lastly, we demonstrate how analyses of partitioned exposures in an MVMR framework helps decipher whether a particular biological mechanism directly contributes to disease risk, offering unique insight for the development of targeted interventions.

## Supplementary Information

Additional file 1: Supplementary tables.

Additional file 2: Supplementary figures.

Additional file 3: Supplementary tables enrichment (STE), dataset containing supplementary results tables from enrichments analysis.

Additional file 4: Supplementary note providing a description and reference of all tables provided in supplementary additional files 1 and 4.

## Data Availability

GWAS summary statistics were accessed via OpenGWAS [89] https://gwas.mrcieu.ac.uk/ for ebi-a-GCST006414 [41], ebi-a-GCST006906 [44], ebi-a-GCST009541 [42], ieu-a- 7 [43], ieu-a- 26 [90], ieu-a- 798 [43], ieu-b- 38 [27], ieu-b- 39 [27], and ieu-b- 40 [40]. Summary data for left ventricular function traits (45) were downloaded from https://personal.broadinstitute.org/ryank/ and early-onset AMD [46] from https://homepages.uni-regensburg.de/~wit59712/earlyamd/winkler_et_al_earlyamd_meta.gz.
