# Supplementary material for: Distinct pathway-based effects of blood pressure and body mass index on cardiovascular traits: comparison of novel Mendelian randomization approaches
Source: Genome Med. 2025 May 15;17:54. doi: 10.1186/s13073-025-01472-2 (PMC12079859; doi:10.1186/s13073-025-01472-2)
Supplement: Supplementary file 2 — Additional file 2: Supplementary figures. [file 13073_2025_1472_MOESM2_ESM.docx]

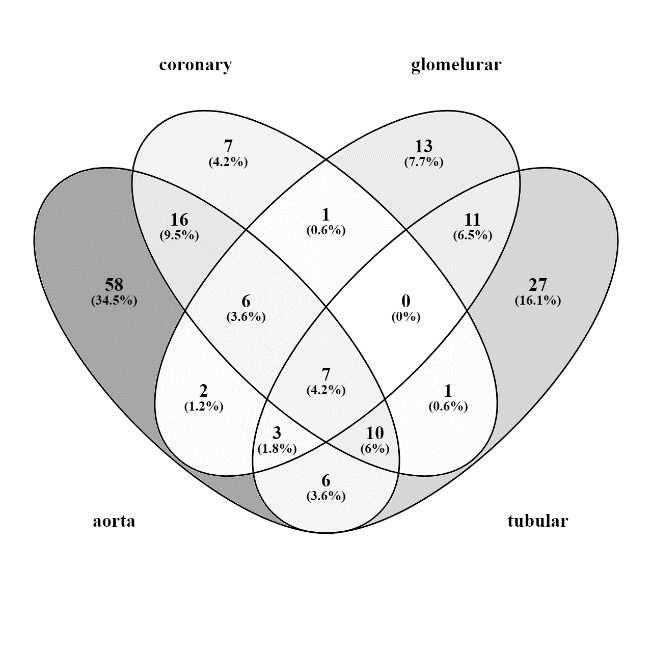

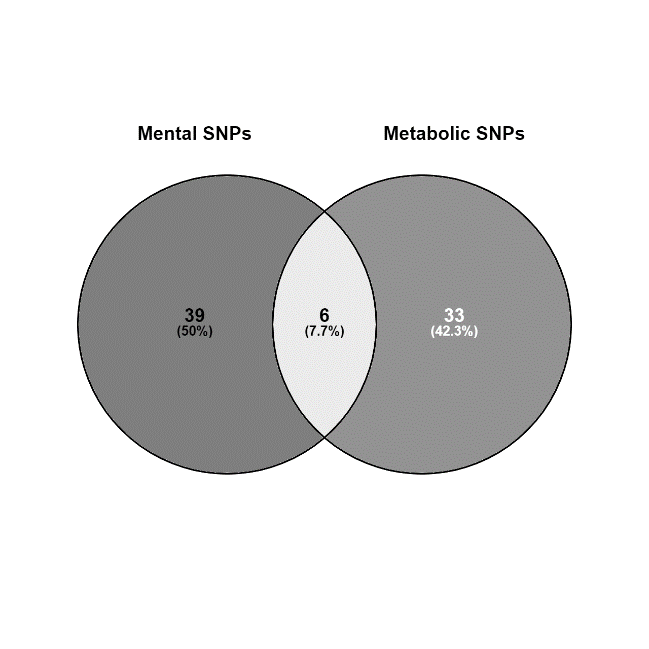

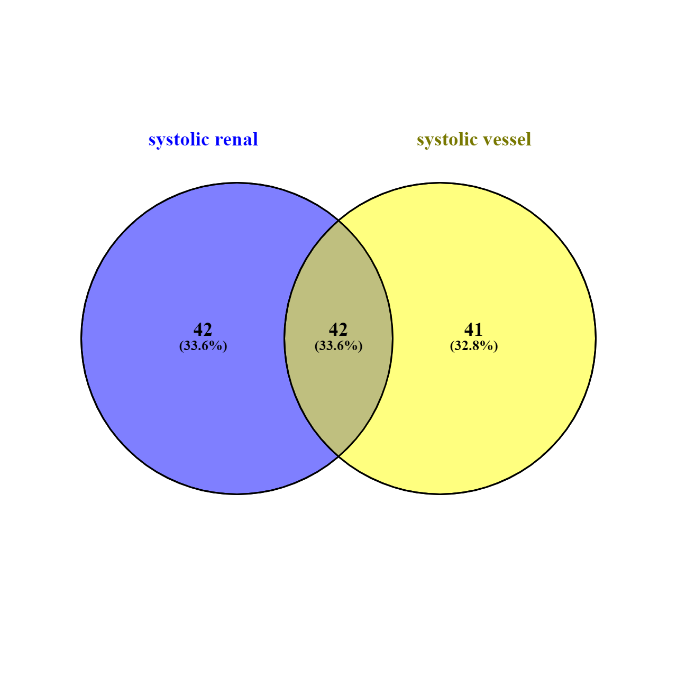

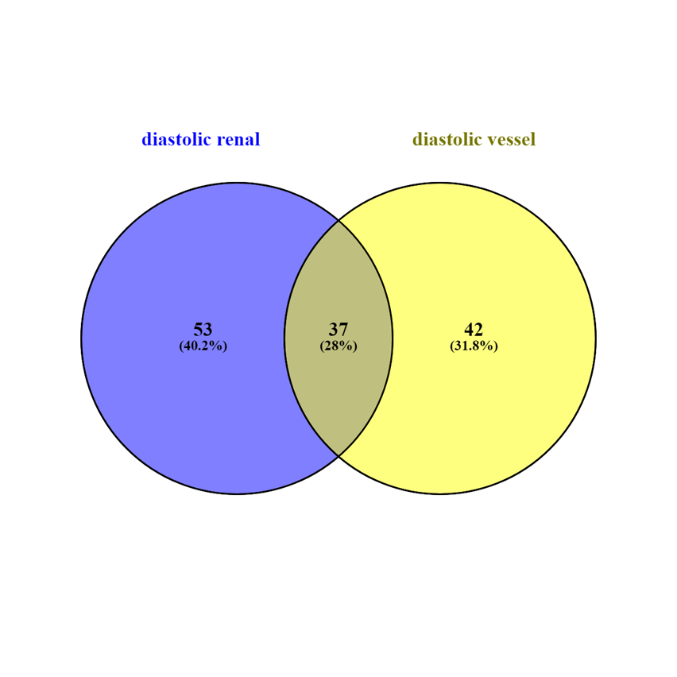


**C**

**D**

**B**

**A**


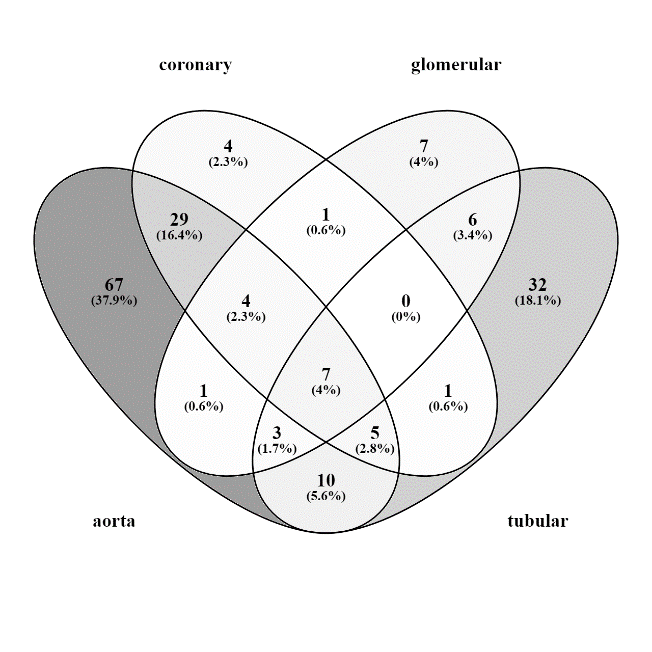

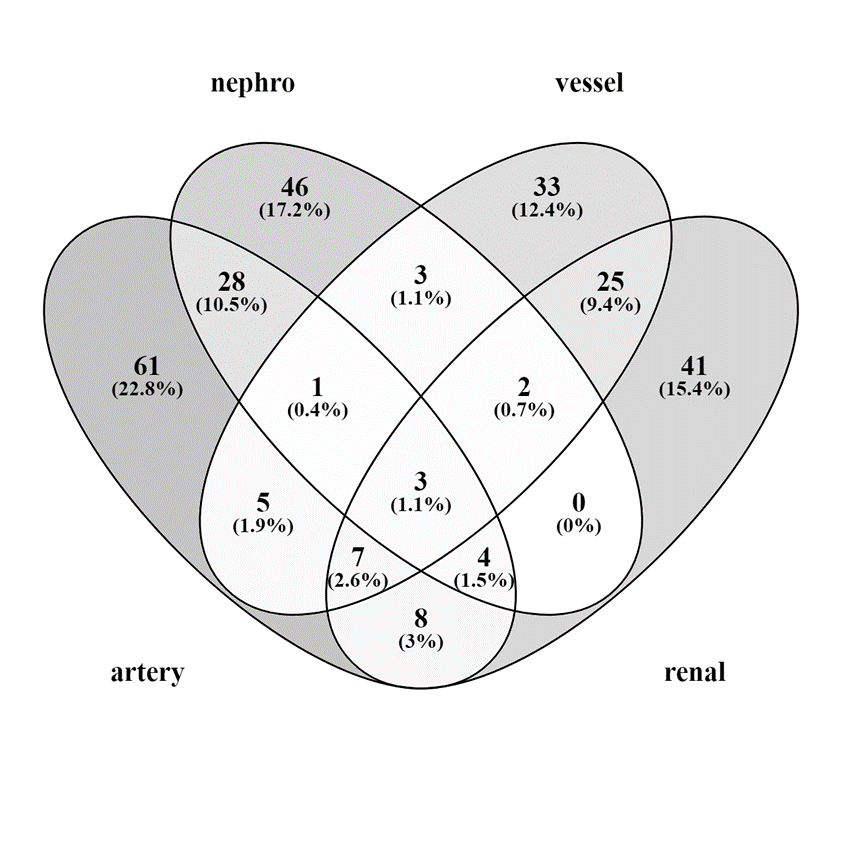


**E**

**F**


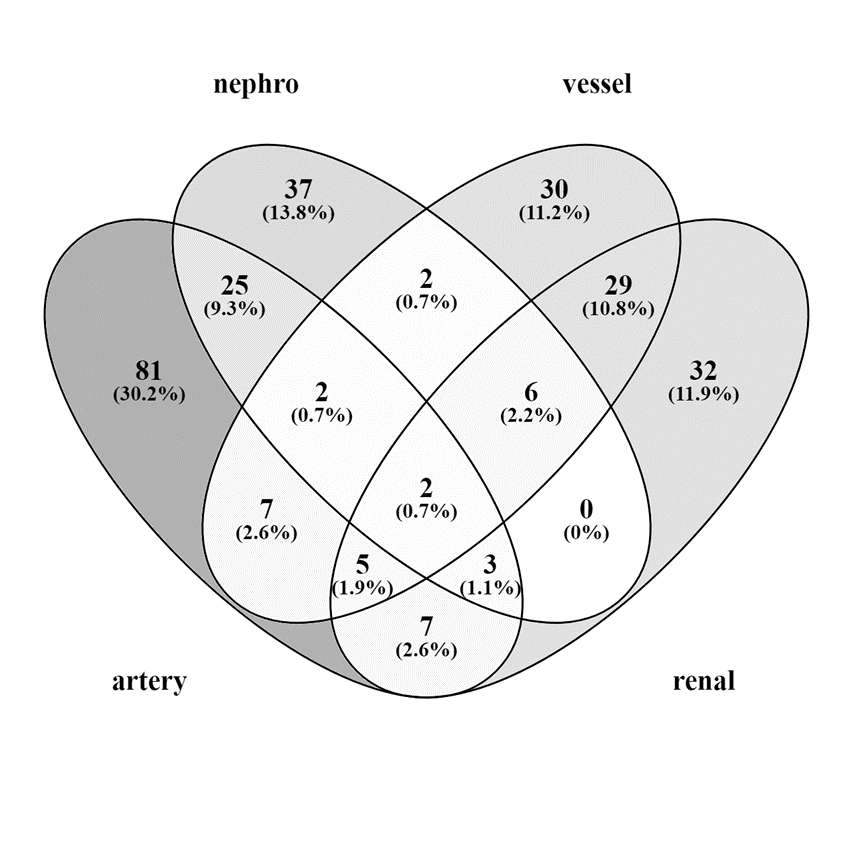

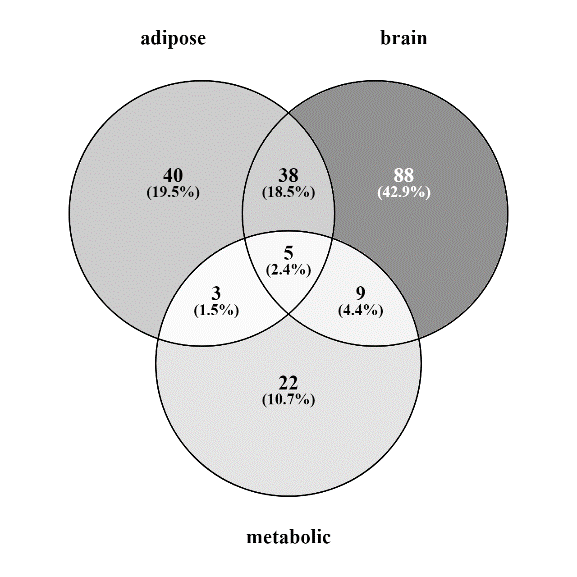

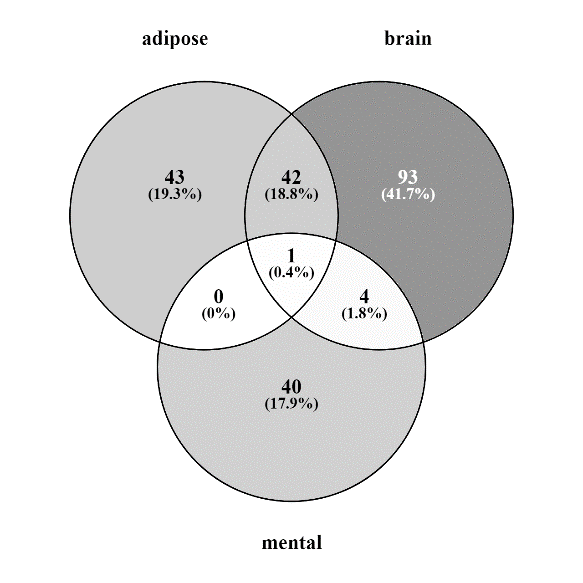


**H**

**I**

**G**

**Figure S1. SNP overlap of Pathway and Tissue partitions.**

A) Pathway partitioned BP SNPs – renal and vessel; B) Pathway partitioned BP SNPs – renal and vessel; C) Pathway partitioned BMI SNPs – mental and metabolic; D) Tissue partitioned diastolic BP SNPs – artery (aorta, coronary) and nephro (glomerular and tubular); E) Tissue partitioned systolic BP SNPs – artery (aorta, coronary) and nephro (glomerular and tubular); F) Pathway partitioned diastolic BP SNPs – renal and vessel vs Tissue partitioned diastolic BP SNPs – artery and nephro; G) Pathway partitioned systolic BP SNPs – renal and vessel vs Tissue partitioned diastolic BP SNPs – artery and nephro; H) Pathway partitioned BMI SNPs – mental vs Tissue partitioned BMI SNPs – adipose and brain; I) Pathway partitioned BMI SNPs –metabolic vs Tissue partitioned BMI SNPs – adipose and brain. We note that different data resources were used in the original identification of BMI-adipose and brain IVs from the Pathway IVs identified in the present study. In H) and I), comparison between Tissue and Pathway partitioned SNPs was made not using just original SNPs but also their proxies (r^2^ > 0.8, 1 Mbp, 1000 Genomes European population) since the coloc SNP dataset contained additional SNPs from UKBB BMI GWAS.


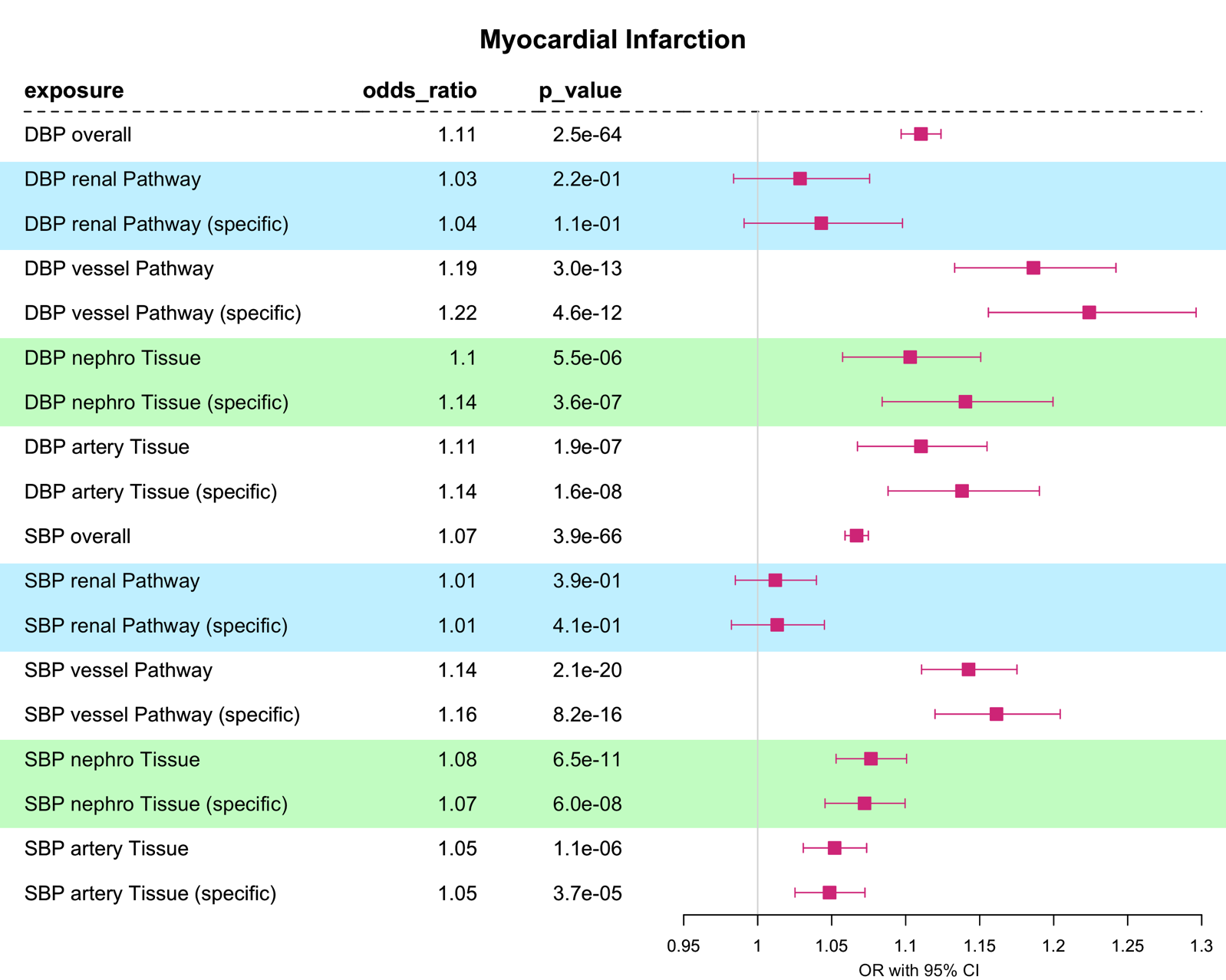


**Figure S2. Myocardial infarction:** one sample Multivariable Mendelian Randomization analysis of the effect of diastolic blood pressure (DBP) and systolic blood pressure (SBP) on MI evaluating the overall trait effect (univariable MR) and Pathway and Tissue partitioned effects (multivariable MR) using all/specific Pathway partitioned IVs (informed by Mendelian disease with abnormalities in the renal or blood vessel system) and all/specific Tissue partitioned IVs (informed by coloc with “nephro” (kidney tissues: glomerular and tubulointerstitial) and “artery“ (aorta and coronary artery) tissues. Effect sizes are scaled to per one SD change in blood pressure.


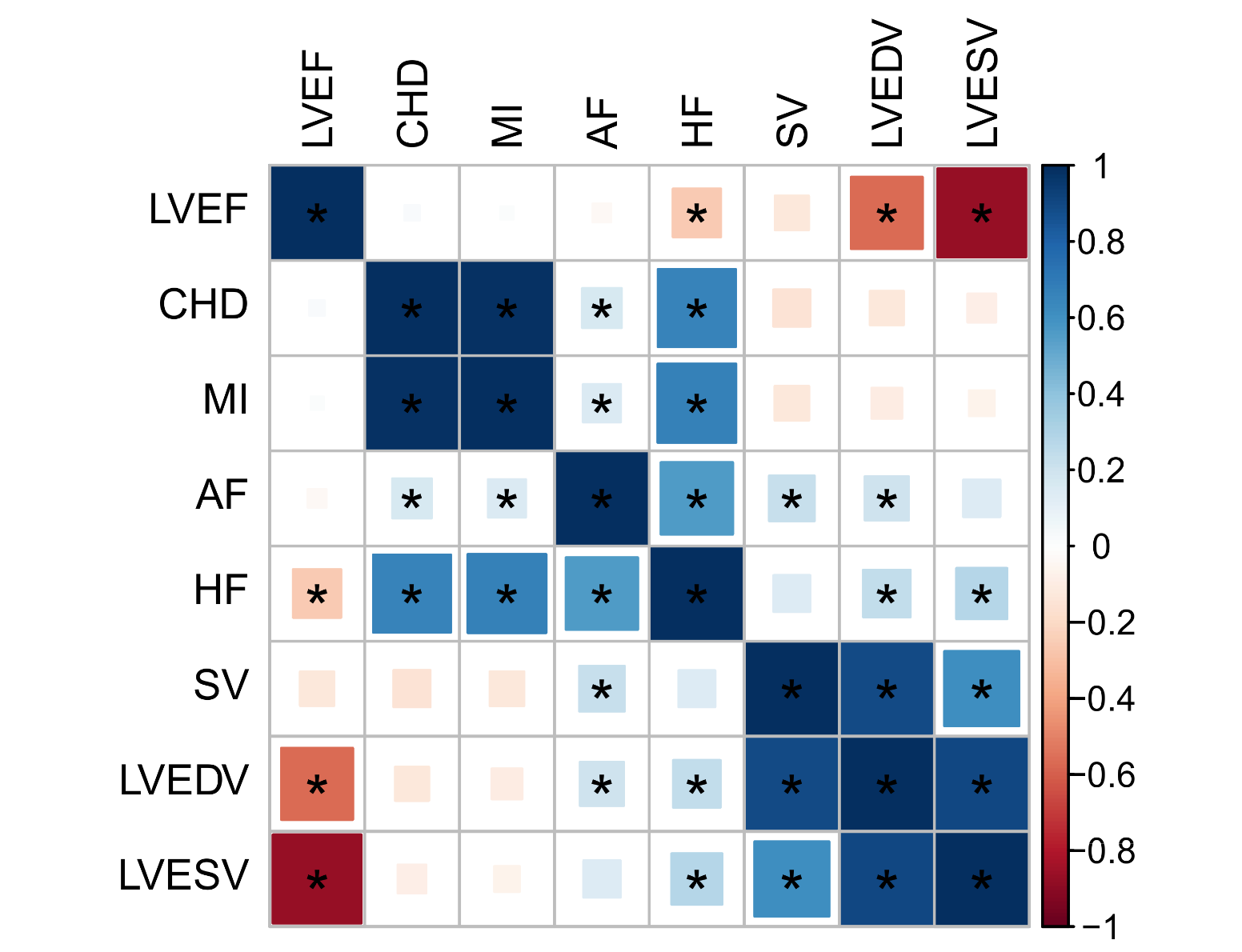


**Figure S3.** Pairwise genetic correlation among cardiac traits estimated using LD score regression in LDSC. Larger area of square filling corresponds to more significant FDR and significant correlation coefficients (FDR < 0.05) are indicated by asterisks. AF - atrial fibrillation, HF - heart failure, CHD - coronary heart disease, MI - myocardial infarction, SV - left ventricle stroke volume, LVEF - left ventricular ejection fraction, LVEDV - left ventricular end-diastolic volume, LVESV - left ventricular end-systolic volume.


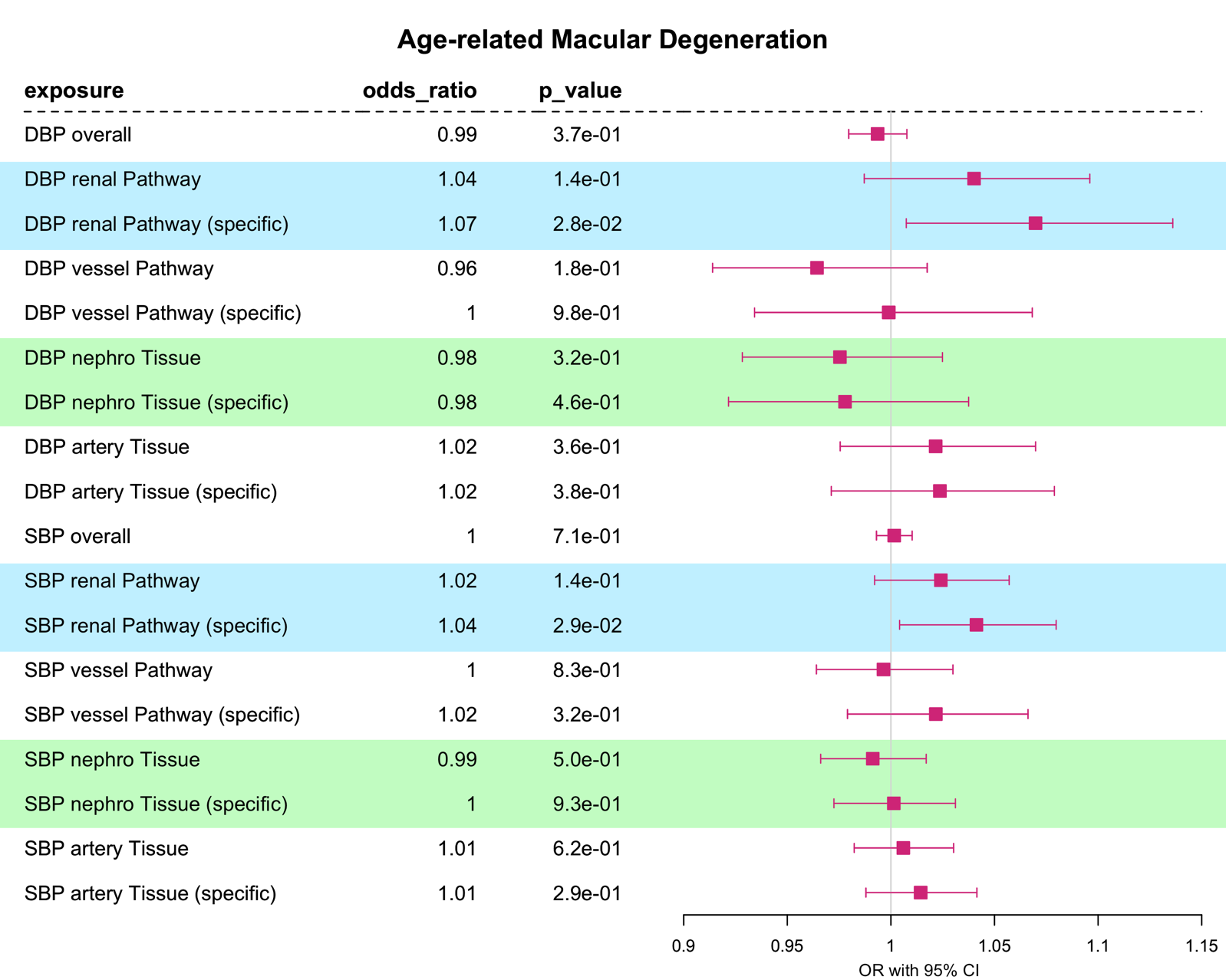


**Figure S4.** **Age-related macular degeneration:** One sample Multivariable Mendelian Randomization analysis of the effect of diastolic blood pressure (DBP) and systolic blood pressure (SBP) on age-related macular degeneration (AMD) evaluating the overall trait effect (univariable MR) and Pathway and Tissue partitioned effects (multivariable MR) using all/specific Pathway partitioned IVs (informed by Mendelian disease with abnormalities in the renal or blood vessel system) and all/specific Tissue partitioned IVs (informed by coloc with “nephro” (kidney tissues: glomerular and tubulointerstitial) and “artery“ (aorta and coronary artery) tissues. Effect sizes are scaled to per one SD change in blood pressure.


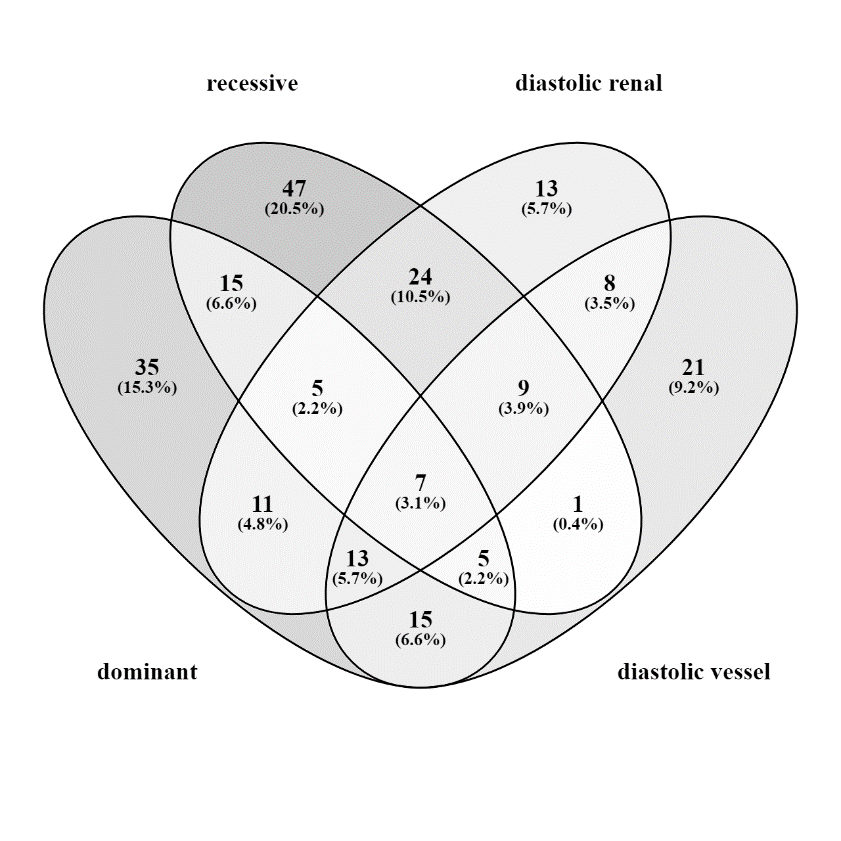


**A**


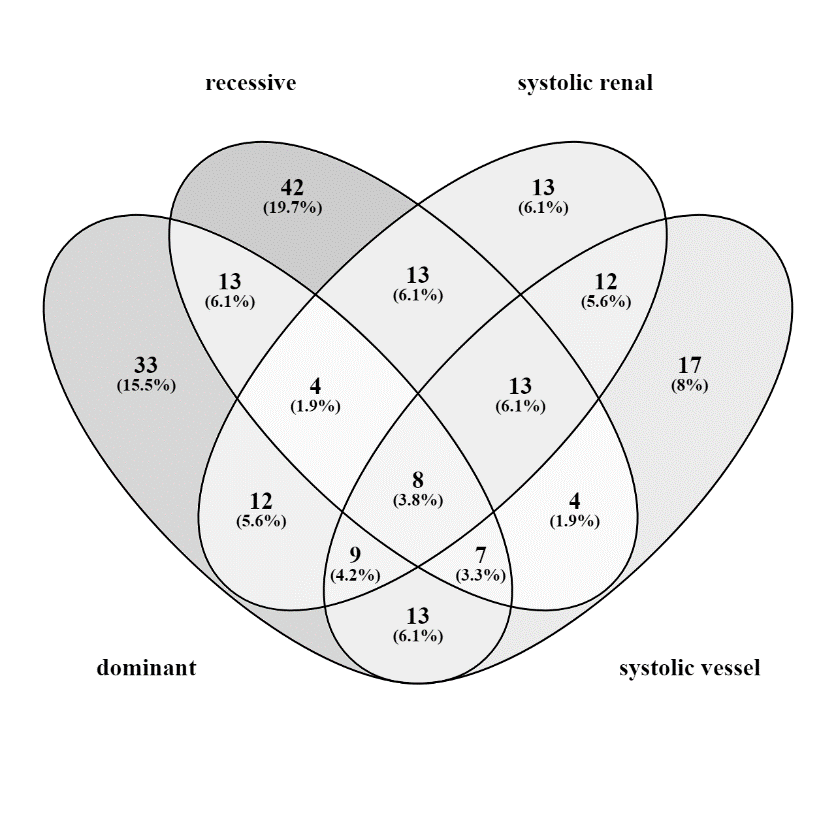


**B**


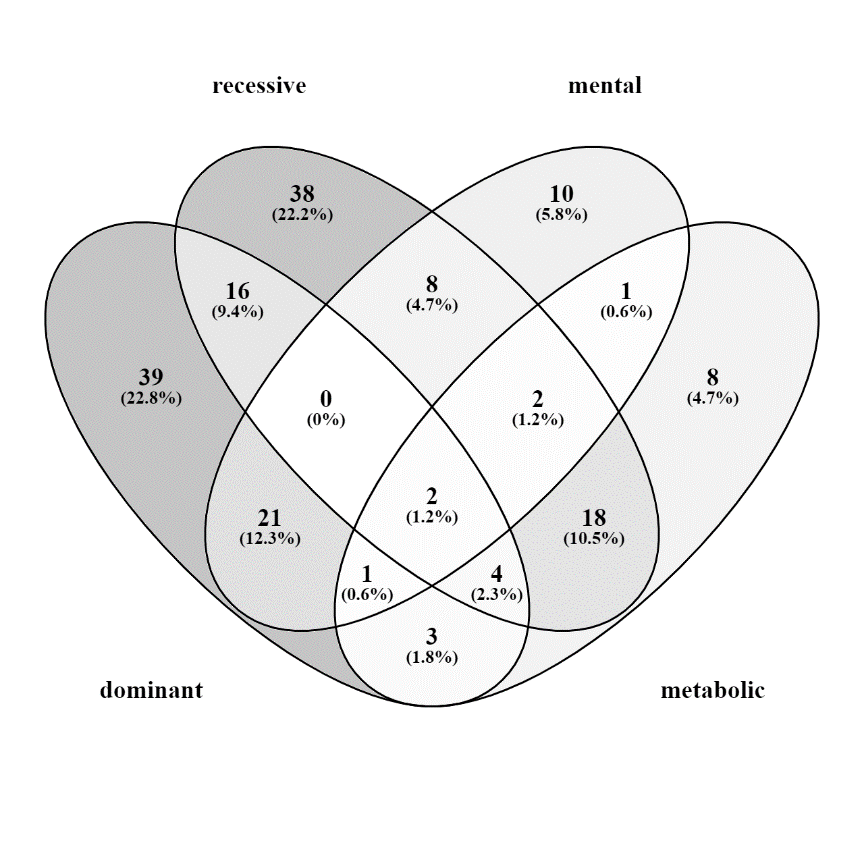


**C**

**Figure S5.** Comparison of SNPs associated with enriched Mendelian disease terms and SNPs associated with Mendelian disease genes inherited in autosomal dominant and autosomal recessive fashion: A) diastolic blood pressure; B) systolic blood pressure; C) body mass index.


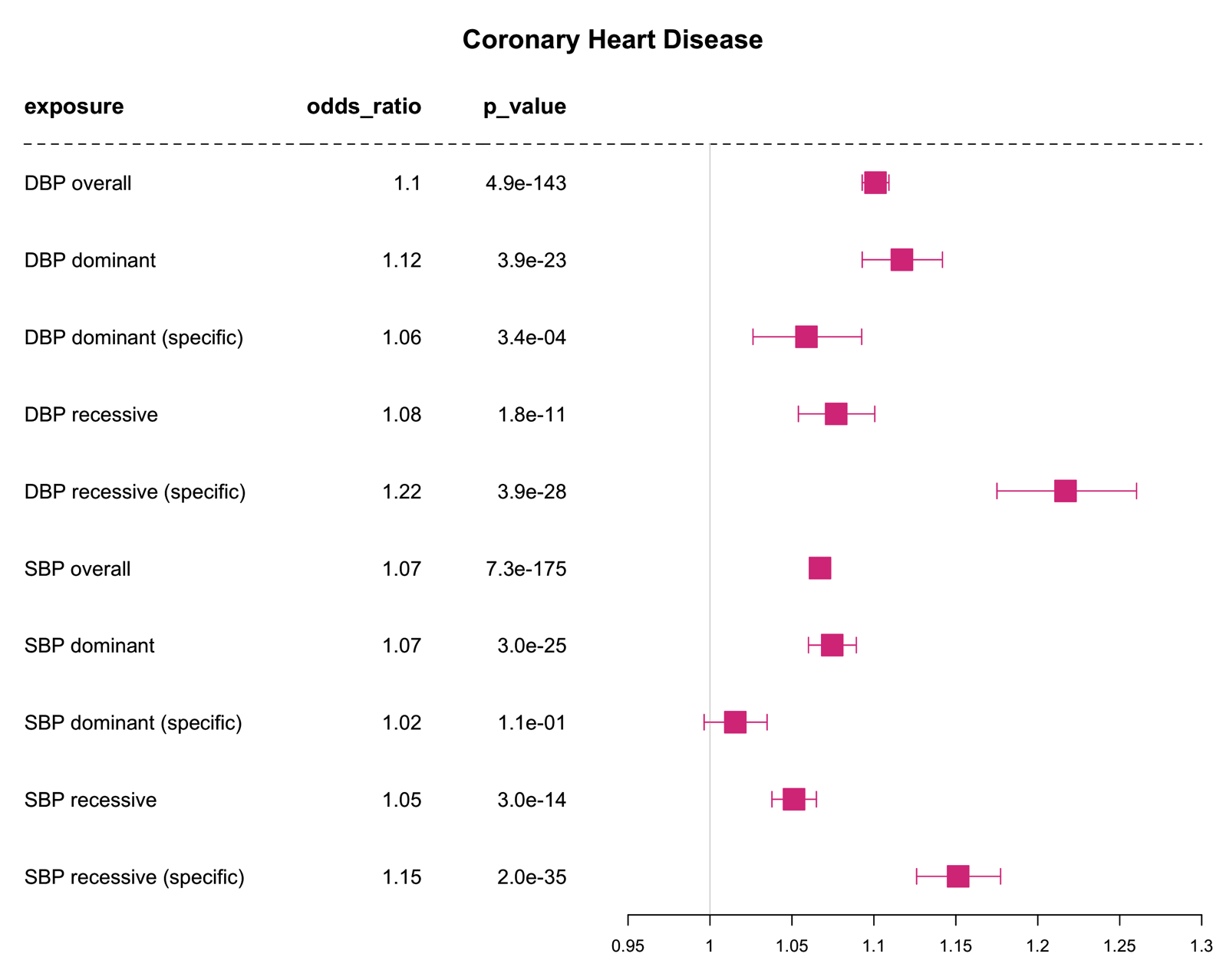


**Figure S6. Coronary heart disease:** One sample Multivariable Mendelian Randomization analysis of the effect of diastolic blood pressure (DBP) and systolic blood pressure (SBP) on CHD using all SNPs, all/specific Pathway-partitioned (disease with autosomal *dominant* or *recessive* inheritance pattern) genetic instruments. Effect sizes are scaled to per one SD change in blood pressure.

**
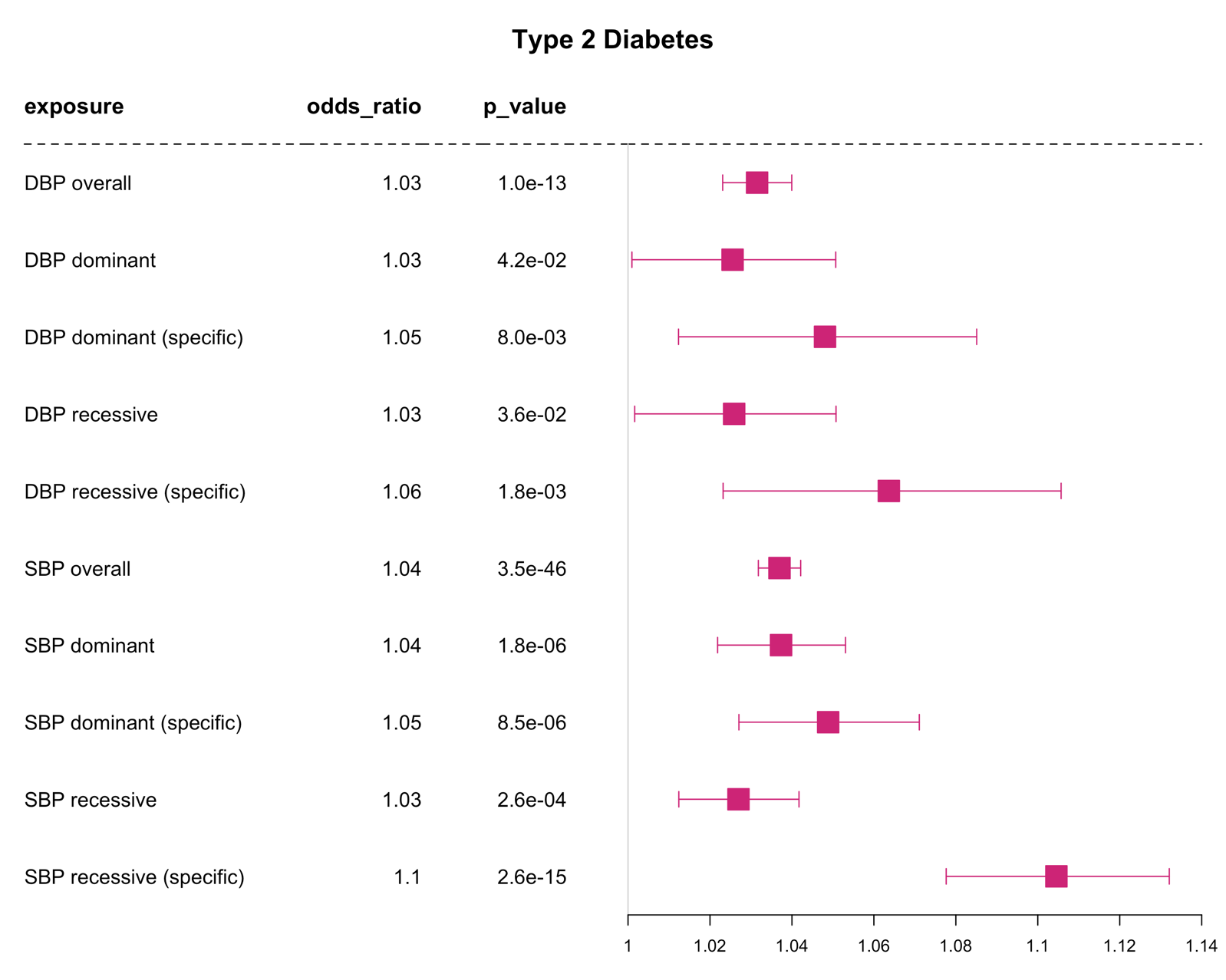
**

**Figure S7. Type 2 diabetes:** One sample Multivariable Mendelian Randomization analysis of the effect of diastolic blood pressure (DBP) and systolic blood pressure (SBP) on T2D using all SNPs, all/specific Pathway-partitioned (disease with autosomal *dominant* or *recessive* inheritance pattern) genetic instruments. Effect sizes are scaled to per one SD change in blood pressure.
