# Supplementary material for: Distinct pathway-based effects of blood pressure and body mass index on cardiovascular traits: comparison of novel Mendelian randomization approaches
Source: Genome Med. 2025 May 15;17:54. doi: 10.1186/s13073-025-01472-2 (PMC12079859; doi:10.1186/s13073-025-01472-2)
Supplement: Supplementary file 4 — Additional file 4: Supplementary note providing a description and reference of all tables provided in supplementary additional files 1 and 4. [file 13073_2025_1472_MOESM4_ESM.docx]

## Supplementary Note

Two Supplementary datasets are provided with this manuscript: Supplementary Tables (1-35) and Supplementary Dataset STE.

Our Supplementary Tables are as follows and are referenced in order in the text:

- Supplementary Table 1 Exposure and outcome datasets used in the study
- Supplementary Table 2 UK Biobank phenotypes used in one sample MR analyses
- Supplementary Table 3 Inheritance mode (autosomal dominant or recessive) of enriched Mendelian disease categories
- Supplementary Table 4 MendelVar enrichment results for DBP using Human Phenotype Ontology
- Supplementary Table 5 MendelVar enrichment results for SBP using Human Phenotype Ontology
- Supplementary Table 6 Summary statistics for all genetic instruments for DBP including Pathway and Tissue annotations
- Supplementary Table 7 Summary statistics for all genetic instruments for SBP including Pathway and Tissue annotations
- Supplementary Table 8 MendelVar enrichment results for BMI using Disease Ontology [slim]
- Supplementary Table 9 Summary statistics for all genetic instruments for BMI including Pathway annotations
- Supplementary Table 10 Colocalisation results for DBP using artery and kidney (nephro) tissues
- Supplementary Table 11 Colocalisation results for SBP using artery and kidney (nephro) tissues
- Supplementary Table 12 One sample MR analyses using all SNPs for DBP and SBP
- Supplementary Table 13 One sample MR (univariable or multivariable) analyses using all or specific Pathway partitioned SNPs for DBP and SBP
- Supplementary Table 14 One sample MR (univariable or multivariable) analyses using all or specific Tissue partitioned SNPs for DBP and SBP
- Supplementary Table 15 Two sample MR analyses using all SNPs for DBP and SBP
- Supplementary Table 16 Two sample MR (univariable) analyses using all or specific Pathway partitioned SNPs for DBP and SBP
- Supplementary Table 17 Two sample MR (univariable or multivariable) analyses using all or specific Tissue partitioned SNPs for DBP and SBP
- Supplementary Table 18 Comparison of absolute effect sizes among the exposures (mean, SE, 95% CI) for SBP, DBP, BMI (all SNPs, and all/ specific Pathway/Tissue partitions).
- Supplementary Table 19 Sensitivity analyses for two sample MR analyses using all SNPs for DBP and SBP - mean F-stats, R2, Cochran's Q, average Cochran's Q, and I2
- Supplementary Table 20 Sensitivity analyses for two sample MR (univariable) analyses using all or specific Pathway partitioned SNPs for DBP and SBP - mean F-stats, R2, Cochran's Q, average Cochran's Q, and I2
- Supplementary Table 21 Sensitivity analyses for two sample MR (univariable) analyses using all or specific Tissue partitioned SNPs for DBP and SBP - mean F-stats, R2, Cochran's Q, average Cochran's Q, and I2
- Supplementary Table 22 One sample MR (univariable or multivariable) analyses using all or specific negative control (mode of inheritance) Pathway partitioned SNPs for DBP and SBP
- Supplementary Table 23 Two sample MR (univariable) analyses using all or specific negative control (mode of inheritance) Pathway partitioned SNPs for DBP and SBP
- Supplementary Table 24 Sensitivity analyses for two sample MR (univariable) analyses using all or specific Pathway partitioned SNPs for DBP and SBP - mean F-stats, R2, Cochran's Q, average Cochran's Q, and I2
- Supplementary Table 25 One sample MR analyses using all SNPs for BMI
- Supplementary Table 26 One sample MR (univariable or multivariable) analyses using all or specific Pathway partitioned SNPs for BMI
- Supplementary Table 27 Two sample MR analyses using all SNPs for BMI
- Supplementary Table 28 Two sample MR (univariable) analyses using all or specific Pathway partitioned SNPs for BMI
- Supplementary Table 29 Replication of Leyden et al. (2022) one sample MR analyses using all SNPs for BMI
- Supplementary Table 30 Replication of Leyden et al. (2022) one sample MR (univariable or multivariable) analyses using all Tissue partitioned SNPs for BMI
- Supplementary Table 31 Replication of Leyden et al. (2022) two sample MR (univariable or multivariable) analyses using all or specific Tissue partitioned SNPs for BMI
- Supplementary Table 32 Sensitivity analyses for two sample MR (univariable) analyses using all or specific Leyden Tissue partitioned SNPs for BMI - mean F-stats, R2, Cochran's Q, average Cochran's Q and I2
- Supplementary Table 33 Sensitivity analyses for two sample MR analyses using all SNPs for BMI - mean F-stats, R2, Cochran's Q, average Cochran's Q and I2
- Supplementary Table 34 Sensitivity analyses for two sample MR (univariable) analyses using all or specific Pathway partitioned SNPs for BMI - mean F-stats, R2, Cochran's Q, average Cochran's Q and I2
- Supplementary Table 35 95% confidence intervals and p-values for observed differences in effect sizes between SNP subsets in all SNP MR analyses determined using random SNP sampling

Our Supplementary Dataset STE T includes the detailed output from Enrichment analysis results for partitioned genetic IVs using two tools: ConsensusPathDB and ToppGen and are as follows:

- STE 1 Pathway renal SNPs for DBP, ConsensusPathDB
- STE 2 Pathway renal SNPs for DBP, ToppGen
- STE 3 Pathway renal SNPs for SBP, ConsensusPathDB
- STE 4 Pathway renal SNPs for SBP, ToppGen
- STE 5 Pathway vessel SNPs for DBP, ConsensusPathDB
- STE 6 Pathway vessel SNPs for DBP, ToppGen
- STE 7 Pathway vessel SNPs for SBP, ConsensusPathDB
- STE 8 Pathway vessel SNPs for SBP, ToppGen
- STE 9 Pathway mental SNPs for BMI, ConsensusPathDB
- STE 10 Pathway mental SNPs for BMI, ToppGen
- STE 11 Pathway metabolic SNPs for BMI, ConsensusPathDB
- STE 12 Pathway metabolic SNPs for BMI, ToppGen
- STE 13 Tissue nephro SNPs for DBP, ConsensusPathDB
- STE 14 Tissue nephro SNPs for DBP, ToppGen
- STE 15 Tissue nephro SNPs for SBP, ConsensusPathDB
- STE 16 Tissue nephro SNPs for SBP, ToppGen
- STE 17 Tissue artery SNPs for DBP, ConsensusPathDB
- STE 18 Tissue artery SNPs for DBP, ToppGen
- STE 19 Tissue artery SNPs for SBP, ConsensusPathDB
- STE 20 Tissue artery SNPs for SBP, ToppGen
- STE 21 Tissue adipose SNPs for BMI [Leyden], ConsensusPathDB
- STE 22 Tissue adipose SNPs for BMI [Leyden], ToppGen
- STE 23 Tissue brain SNPs for BMI [Leyden], ConsensusPathDB
- STE 24 Tissue brain SNPs for BMI [Leyden], ToppGen
